# Levosimendan inhibits disulfide tau oligomerization and ameliorates tau pathology in Tau^P301L^-BiFC mice

**DOI:** 10.1038/s12276-023-00959-5

**Published:** 2023-03-13

**Authors:** Sungsu Lim, Seulgi Shin, Yoonsik Sung, Ha Eun Lee, Kyu Hyeon Kim, Ji Yeon Song, Gwan-Ho Lee, Hira Aziz, Nataliia Lukianenko, Dong Min Kang, Nicolette Boesen, Hyeanjeong Jeong, Aizhan Abdildinova, Junghee Lee, Byung-Yong Yu, Sang Min Lim, Jun-Seok Lee, Hoon Ryu, Ae Nim Pae, Yun Kyung Kim

**Affiliations:** 1 Center for Brain Disorders, Brain Science Institute, Korea Institute of Science and Technology (KIST), Seoul, 02792 Republic of Korea; 2 Division of Bio-Medical Science & Technology, KIST School, Korea University of Science and Technology (UST), Seoul, 02792 Republic of Korea; 3 Advanced Analysis Center, Korea Institute of Science and Technology (KIST), Seoul, 02792 Republic of Korea; 4 Department of Life Sciences, Korea University, Seoul, 02841 Korea; 5 Boston University Alzheimer’s disease Research Center and VA Boston Health care System, Boston, MA 02130 USA; 6 Department of Pharmacology, Korea University College of Medicine, Seoul, 02792 Republic of Korea; 7 Boston University Alzheimer’s disease Research Center and Department of Neurology, Boston University School of Medicine, Boston, MA 02118 USA

**Keywords:** Alzheimer's disease, Drug development

## Abstract

Tau oligomers play critical roles in tau pathology and are responsible for neuronal cell death and transmitting the disease in the brain. Accordingly, preventing tau oligomerization has become an important therapeutic strategy to treat tauopathies, including Alzheimer’s disease. However, progress has been slow because detecting tau oligomers in the cellular context is difficult. Working toward tau-targeted drug discovery, our group has developed a tau-BiFC platform to monitor and quantify tau oligomerization. By using the tau-BiFC platform, we screened libraries with FDA-approved and passed phase I drugs and identified levosimendan as a potent anti-tau agent that inhibits tau oligomerization. ^14^C-isotope labeling of levosimendan revealed that levosimendan covalently bound to tau cysteines, directly inhibiting disulfide-linked tau oligomerization. In addition, levosimendan disassembles tau oligomers into monomers, rescuing neurons from aggregation states. In comparison, the well-known anti-tau agents methylene blue and LMTM failed to protect neurons from tau-mediated toxicity, generating high-molecular-weight tau oligomers. Levosimendan displayed robust potency against tau oligomerization and rescued cognitive declines induced by tauopathy in the Tau^P301L^-BiFC mouse model. Our data present the potential of levosimendan as a disease-modifying drug for tauopathies.

## Alzheimer’s disease: A tool for preventing toxic tangles

The discovery that an already approved drug can disrupt tau protein aggregation in cultured cells and live mice could guide the development of new therapies for Alzheimer’s. Tau plays an important role in organizing the internal structure of neurons, but in neurodegenerative disease is seen to form abnormal assemblies that are toxic to cells. Researchers led by Ae Nim Pae and Yun Kyung Kim at the Korea Institute of Science and Technology, Seoul, South Korea have devised an assay that allowed them to identify agents that actively interfere with this process. Levosimendan, a drug approved for heart failure, broke up tau aggregates in cell culture and reduced cognitive symptoms in a mouse model of tau-induced neurodegeneration. Further investigation of levosimendan’s mode of action could yield interventions that slow or prevent Alzheimer’s progression.

## Introduction

Tau is naturally an extremely soluble protein and contains many positively charged lysine residues^1^. The lysine residues play a role in binding negatively charged microtubules^2^. Upon binding, tau stabilizes microtubules and promotes microtubule assembly, which is critical for axonal outgrowth^3,4^. Under pathological conditions, the charge balance between tau and microtubules is disrupted, and tau dissociates from the microtubules^5^. The dissociated tau becomes a substrate that is susceptible to cytosolic enzymes, which modify the states of tau phosphorylation, acetylation, or proteolytic cleavage^6,7^. Chemically and structurally modified tau aggregates and accumulates in neurons. Intraneuronal deposits of tau fibrils (PHFs and NFTs) are the pathological hallmark in a group of neurodegenerative diseases called tauopathies. Tauopathies include not only Alzheimer’s disease (AD) but also fronto-temporal dementia (FTD), progressive supranuclear palsy, corticobasal degeneration, and chronic traumatic encephalopathy^8,9^. Due to the implication of tau in neurodegenerative disorders, tau has become an important therapeutic target^10^. However, progress has been slow because the pathology of tau is not well understood.

Until recently, neurotoxic tau species were presumed to be filamentous tau (PHFs and NFTs), accumulated in the brains of patients with AD. However, mounting evidence suggests that soluble tau oligomers, rather than fibrils, are the neurotoxic species responsible for neuronal cell death^11,12^. Tau oligomers are soluble forms of tau aggregates ranging from dimers to prefibrillar aggregates^13^. In AD brains, tau oligomers are detected in the early stage of pathogenic cascades, and the level of tau oligomers is correlated with synaptic dysfunction and neuronal cell loss, rather than the levels of NFTs^14–16^. Evidence has also suggested that tau oligomers are transmittable between neurons, spreading the disease in the brain^17,18^. Due to their relatively small size, tau oligomers can penetrate into cells and initiate nascent tau aggregation, providing structural seeds for aggregation^19^. The formation of insoluble filaments (PHFs and NFTs) might be a survival mechanism for neurons to quarantine toxic tau species from the cytosol^20^. Accordingly, prevention of tau oligomerization or elimination of oligomers has become an important therapeutic strategy for developing drugs to treat AD.

The most studied tau aggregation inhibitor is methylene blue (MB), a blue dye that has been widely used in various industrial and research settings. In 2008, Wischik et al. first reported the anti-tau activity of MB^21^. In their study, MB disrupted the stability of NFTs isolated from AD patients. In 2013, Zweckstetter et al. reported the inhibitory mechanism of MB on tau aggregation. In their study, MB inhibited tau aggregation through the oxidation of tau cysteine residues, converting tau into aggregation-incompetent monomers^22^. However, in conflict, data also show that MB increases tau dimers through the same cysteine-oxidation mechanism^23^. Full-length tau contains two cysteine residues (C291 and C322) in the microtubule-binding domain. Intramolecular disulfide bond formation generates a compact monomer resistant to tau aggregation, and intermolecular disulfide bonds facilitate the aggregation cascade by generating structurally stable tau oligomers^24^. In 2019, Takashima et al. reported that MB inhibited the formation of tau fibrils but not tau oligomers^25^. The mechanism of action for tau pathology remains unclear, but the reduced form of MB, LMTM (also known as TauRx0237 or LMT-X), has completed phase III clinical trials for the treatment of AD. In the first phase III trial, LMTM failed to ameliorate cognitive decline in patients with mild to moderate AD^26,27^. Although it failed in AD treatment, LMTM is still in clinical trials for the treatment of FTDs.

**Fig. 1:**
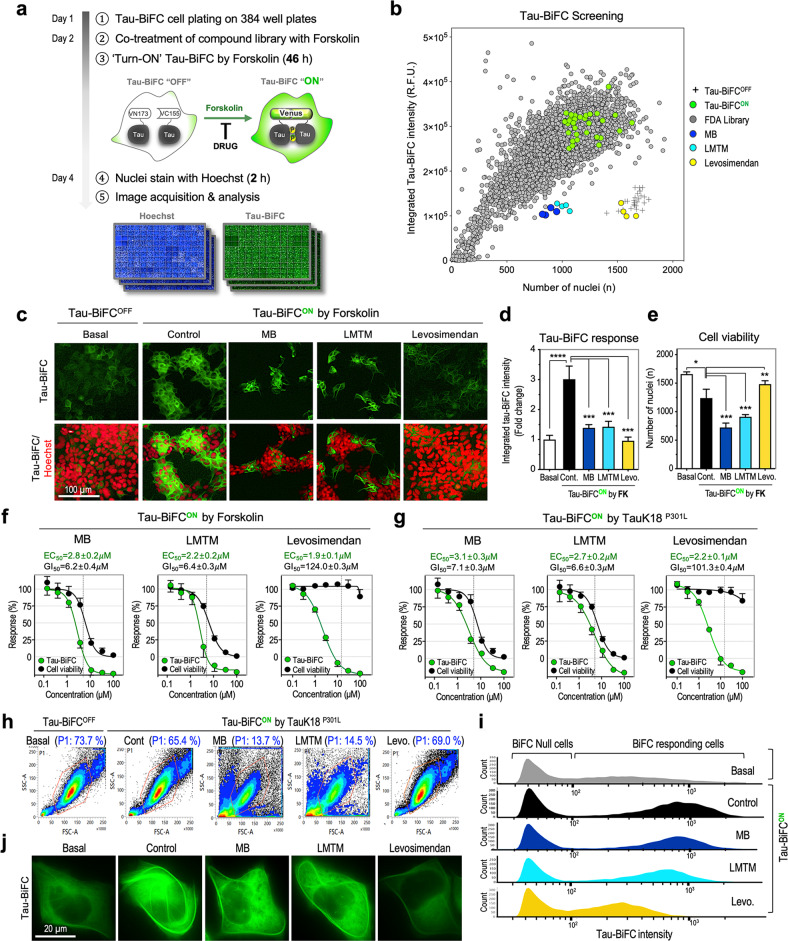
Screening the FDA-approved & Passed Phase I Drug Library revealed levosimendan as a new anti-tau agent. **a** Schematic representation of tau-BiFC screening of FDA-approved & Passed Phase I Drug Library. For tau-BiFC screening, Tau-BiFC cells were treated with each 1,018 library compound (10 μM) in the presence of forskolin (FK). MB and LMTM (3 μM) were used as positive controls. **b** Scatterplot of 1,018 FDA-approved drugs screened. Quadruplicate results of a single drug were plotted with the BiFC intensity on the Y-axis and the number of nuclei on the X-axis. Crosses indicate BiFC^OFF^-basal, and green dots indicate BiFC^ON^-control. One selective drug, levosimendan (yellow), was plotted with MB (blue) and LMTM (cyan). **c** BiFC fluorescence images of tau-BiFC cells treated with MB, LMTM, or levosimendan in the presence of FK. Nuclei were counterstained with Hoechst and indicated in red color. Scale bar, 100 μm. Quantification of tau-BiFC responses (**d**) and cell viabilities (**e**). One-way ANOVA with Dunnett’s multiple-comparisons test was performed; **p < 0.01, ***p < 0.001, compared with control. Dose‒response curves of MB, LMTM, and levosimendan with EC_50_ and GI_50_ values. In the presence of FK (**f**) or tauK18^P301L^ (**g**), tau-BiFC cells were treated with each drug at various concentrations. After 48 h of treatment, tau-BiFC responses and the number of nuclei were analyzed. Prism’s nonlinear regression analysis was used to measure the EC_50_ and GI_50_ values. **h**, **i** Flow cytometry analysis of tau-BiFC cells cotreated with MB, LMTM (5 μM), or levosimendan (15 µM) with TauK18^P301L^. **h** P1 population indicates mononuclear cells gated with a red line based on the level of side scatter (SSC) and forward scatter (FSC), excluding cell debris or dead cells. The percentage of P1 indicates the ratio of healthy, mononuclear cells out of total cells. **i** BiFC fluorescence histograms of P1 cell populations. **j** High magnification images of a representative tau-BiFC cell treated with MB, LMTM, and levosimendan. Scale bar, 20 μm.

Tau aggregation is a complicated multistep process that is controlled by a number of cellular enzymes. Therefore, in many cases, tau aggregation inhibitors that show strong inhibitory effects in in vitro tau aggregation assays are not effective in cellular systems. Therefore, the mechanism of action of tau-targeted drugs should be evaluated in a cellular system, not in test tubes using purified tau. In the intracellular space, diverse tau modifications occur simultaneously, changing its physiological properties; phosphorylation reduces the microtubule binding affinity of tau^28,29^, acetylation prevents its ubiquitin-mediated degradation^30^, and dityrosine or disulfide-bond formation promotes tau aggregation by generating covalently linked tau oligomers^24,31^. In this regard, our group has developed a tau-BiFC platform to monitor tau oligomerization in living cells and in the brains of mice^32,33^. In the tau-BiFC system, nonfluorescent N- and C-terminal compartments of the Venus protein are fused to tau, and Venus fluorescence turns on only when tau assembles together (Fig. 1a). By using the tau-BiFC cell model, we have characterized prion-like tau oligomers^19^ and investigated pathological tau modification^34–36^.

In this study, by using the tau-BiFC platform, we screened the FDA-approved & Passed Phase I Drug Library and identified levosimendan as a new anti-tau agent.

## Materials and methods

### Source of chemicals

FDA-approved & Passed Phase I Drug Library was purchased from Selleckchem. Forskolin and MB were purchased from Sigma, and leucomethylene blue mesylate (LMTM) was purchased from MedChemExpress. Levosimendan and its metabolites, OR-1855 and OR-1896, were purchased from Toronto Research Chemicals Inc. Synthesis and characterization of ^14^C-levosimendan were conducted by Curachem, Inc. according to a previously described protocol^37^. The characterizations of ^14^C-levosimendan are provided in the Supplementary Materials.

### Preparation of tauK18 fragments

TauK18 and tauK18^P301L^ fragments were expressed and purified according to previously described protocols^19,38^. 6xHis-tagged tauK18 and tauK18^P301L^ were expressed in *E. coli* BL21 (DE3) and purified by using Ni-NTA (QIAGEN) column chromatography. The purified protein was dissolved in PBS (pH 7.4). Peptide synthesis and characterization of each repeat domain (R1-R4) were conducted by GL Biochem. ESI-MS characterization of the peptides is provided in the Supplementary Materials.

### Tau-BiFC cell culture and treatment with FK or tauK18P301L

HEK293 Tau-BiFC cells were maintained in Dulbecco’s modified Eagle medium (DMEM) supplemented with 10% fetal bovine serum (FBS), 100 units/mL penicillin, 100 µg/mL streptomycin and 100 µg/mL Geneticin (G418) in a humidified atmosphere containing 5% CO_2_ at 37 °C. Tau-BiFC cells were activated by treatment with FK (30 μM) or tauK18^P301L^ (5 μg/mL).

### Screening and high-content image analysis of a 1018 FDA-approved & Passed Phase I Drug Library

For screening of FDA-approved & Passed Phase I Drug Library, tau-BiFC cells were plated on µ-clear 384-well plates. The next day, the cells were cotreated with forskolin and 1,018 library compounds at a concentration of 10 µM. MB and LMTM (3 µM) were used as positive controls. After 46 h, nuclei were counterstained with Hoechst (Invitrogen). BiFC (λ_ex_ = 460–490 nm and λ_em_ = 500–550 nm) and Hoechst (λ_ex_ = 355–385 nm and λ_em_ = 430–500 nm) fluorescence images were acquired automatically by using Operetta® (PerkinElmer). BiFC fluorescence intensity and the number of nuclei were quantified using Harmony 3.1 software (PerkinElmer). Integrated tau-BiFC intensity represents the area of selected cell X mean fluorescence intensity. The means and standard deviations (S.D.) of BiFC fluorescence intensities and the number of nuclei were plotted using GraphPad Prism.

### Flow cytometry analysis

Tau-BiFC cells grown in 6-well plates were treated with MB, LMTM (5 µM), or levosimendan (15 µM), followed by treatment with tauK18^P301L^ (5 µg/mL). After 24 h, BiFC cells were collected and subjected to flow cytometry analysis using a BD FACSLyric^TM^ cytometer (BD Biosciences). A total of 50,000 events were acquired per sample. All samples were gated using the same gating tree and gate positions: side scatter area (SSC-A) vs. forward scatter area (FSC-A) for viable and singlet cell populations. BiFC fluorescence was excited by a 488 nm wave laser and collected through a 527/32 bandpass filter.

### Preparation of Tau-BiFC cell lysates

Tau-BiFC cells grown in 6-well plates were treated with MB, LMTM (0.5, 1.5, 5 µM), or levosimendan (5, 15, 45 µM) followed by tauK18^P301L^ (5 µg/mL) or FK (30 µM) activation. After 36 h, tau-BiFC cells were washed with PBS and lysed in CelLytic M lysis reagent (Sigma) containing protease/phosphatase inhibitor cocktail (Sigma).

### Tau-immunoblot analysis of reducing and nonreducing SDS‒PAGE gels

For reducing SDS‒PAGE analysis, cell or brain lysates were mixed with 4x Laemmli buffer containing 10% β-mercaptoethanol (BME) and boiled at 97 °C for 5 min. For nonreducing SDS‒PAGE analysis, cell or brain lysates were mixed with 4x Laemmli buffer without BME. For immunoblot analysis, 10 μg of each lysate was separated on a 10% SDS–PAGE gel and transferred to a PVDF membrane. The levels of total tau and phosphorylated tau were detected by anti-tau antibodies against 2B11 (IBL), Tau5 (Abcam), pSer199 (Abcam), pSer396 (Abcam), and pThr205 (Abcam). For immunoblot analysis of tau kinases, the levels of total and phosphorylated tau kinase were detected by anti-GSK3β (Abcam), anti-ERK1/2 (Cell Signaling), anti-P35/P25 (Cell Signaling), and anti-CDK5 (Abcam) antibodies. β-actin (Abcam) and GAPDH (Cell Signaling) were used as loading controls. Band intensity was quantified using ImageJ software (NIH). All data were normalized to β-actin.

### Primary neuron culture and preparation of the lysates

Primary hippocampal neurons were isolated from Day 18 embryonic Sprague‒Dawley rat brains as described previously^39^. The neurons were seeded at a density of 3.5 × 10^5^ cells per well on a poly-D-lysine-coated 6-well plate and maintained in neurobasal medium at 37 °C in a humidified atmosphere of 5% CO_2_. The neurobasal medium contained 2% B27 supplement, 0.5 mM GlutaMAX, 100 units/mL penicillin, and 100 μg/mL streptomycin. Every 3 days, 50% of the medium was replaced with fresh neurobasal medium. At DIV10, neurons were incubated with MB, LMTM (3 µM), or levosimendan (10 µM) in the presence of tauK18^P301L^ (10 µg/mL) for 48 h. Then, the neurons were washed with PBS and lysed in CelLytic M lysis reagent (Sigma) containing protease/phosphatase inhibitor cocktail (Sigma).

### NeuO staining and analysis of neurite lengths

At DIV10, neurons grown on a 96-well plate were treated with MB, LMTM (3 µM), or levosimendan (10 µM) followed by tauK18^P301L^ activation. After 48 h, the neurons were stained with NueO (50 nM), a neuron-selective probe^40^. NeuO fluorescence (λ_ex_ = 460–490 nm and λ_em_ = 518–580 nm) images were acquired by using Operetta® (PerkinElmer). The number of neurons and total lengths of neurites were quantified by Harmony 3.1 software (PerkinElmer). Error bars represent the standard deviations (S.D.) of four replicate experiments.

### TauK18 oligomerization assay in vitro

To induce tau oligomerization, tauK18 (0.5 mg/mL dissolved in PBS, pH 7.4) was incubated with each drug in the presence of DTT (100 μM) and heparin (0.1 mg/mL) at RT for 5 h with vigorous shaking. Then, tau oligomers were separated on 4–20% SDS‒PAGE gels under reducing and nonreducing conditions and visualized by Coomassie blue staining.

### In vitro tau aggregation and disaggregation assays

To evaluate the inhibitory effect of drugs on tau aggregation, tauK18 protein (0.5 mg/mL) was incubated with each drug at various concentrations in the presence of DTT (100 μM) and heparin (0.1 mg/mL) at 37 °C for 5 days with vigorous shaking (220 rpm). For the tau disaggregation assay, the preformed aggregation mixture of tauK18 was incubated with each drug at 37 °C for 4 days with vigorous shaking (220 rpm). On the final day of incubation, the level of β-sheet aggregates was evaluated by thioflavin S (ThS) assay. For the ThS assay, 5 μL of each mixture was transferred to a black 384-well plate with 45 μL of PBS containing 10 μM ThS. ThS fluorescence (λ_ex_ = 430 nm, and λ_em_ = 500 nm) was measured by using a Flexstation2 spectrophotometer (Molecular Devices).

### Transmission electron microscopy (TEM)

Samples were placed onto carbon-coated copper electron microscopy grids and then negatively stained with 2% (w/v) aqueous uranyl acetate for 1 min. To image the tau filaments (Fig. 3d), the grids were observed using a JEM-1011 transmission electron microscope (JEOL) at an acceleration voltage of 80 kV. To image the tau filaments and oligomers (Figs. 3f and 4e), the grids were observed using a Tecni G2 F20 transmission electron microscope (FEI) at an acceleration voltage of 120 kV. Eight to twelve random images from each experimental condition were captured by the operator as a blind observer.

### Autoradiography of ^14^C-levosimendan

TauK18 (1 mg/mL, 72 μM) was incubated with ^14^C-levosimendan (720 μM) in PBS containing 5% DMSO at RT for 2 h. Then, the mixture was separated on a 15% SDS–PAGE gel under nonreducing conditions and stained with Coomassie blue. For autoradiography, the SDS–PAGE gel was transferred to a PVDF membrane and scanned by Typhoon FLA 7000 IP (GE Healthcare).

### MALDI-TOF analysis of R1-R4 peptides

Each repeat domain (R1, R2, R3, R4, R2-C291S or R3-C322S, 150 μM) was incubated with levosimendan (molar ratio 1:10 of a tau repeat domain: levosimendan) at RT for 37 h. The samples were mixed with matrix solution (10 mg mL^−1^ sinapinic acid in 0.1% (v/v) trifluoroacetic acid/CAN) at a ratio of 1:1. The mixtures were directly spotted onto the MALDI target and dried. Mass spectra were acquired in reflection/linear positive ion mode in the *m/z* range of 2000–100,000 using an Ultraflex III TOF/TOF mass spectrometer controlled by Flex Control 3.0 (Bruker Daltonics). The default operating conditions were as follows: ion source 1, 25.0 kV; ion source 2, 23.0 kV; lens voltage, 6.0 kV; laser repetition rate = 100 Hz. All spectra were generated automatically in the instrument software and based on averaging 1000 shots from 10 nonoverlapping positions (100 shots/position).

### ^1^H NMR spectrometry of levosimendan with *N*-acetyl-L-cysteine methyl ester

NMR analysis was performed at 25 °C and recorded in δ units relative to deuterated solvent as an internal reference using a 400 MHz NMR instrument (Bruker). Deuterated phosphate-buffered saline (d-PBS) was prepared at a concentration of 50 mM (D2O + H2O, 1:1, v/v). Levosimendan (50 mg, 180 μM, 1 equivalent) reacted with *N*-acetyl-L-cysteine methyl ester (38 mg, 210 μM, 1.2 equivalent) in 10 mL of d-PBS/DMSO-d6 (1:1, v/v) at room temperature, and the reaction was checked at various time points. For the reversibility study, the reaction mixture was diluted ten times with d-PBS/DMSO-d6 (1:1, v/v) and monitored at room temperature.

### Animal studies

#### Tau^P301L^-BiFC mice

Tau^P301L^-BiFC mice were bred and maintained as described previously^33^. Tau^P301L^-BiFC mice were bred with C57BL/6N mice and maintained in pathogen-free facilities. Their heterogeneous offspring and wild-type littermates were used in this study. All mice were allocated randomly for experiments, but groups were counterbalanced for animal sex and group average body weight. Animal protocols followed the principles and practices outlined in the approved guidelines by the Institutional Animal Care and Use Committee of the Korea Institute of Science and Technology. All animal experiments were approved by the Korea Institute of Science and Technology.

#### Drug administration to Tau^P301L^-BiFC mice

For drug administration, LMTM and levosimendan were dissolved in PBS containing 40% polyethylene glycol (PEG; Sigma). Nine-month-old Tau^P301L^-BiFC mice were intraperitoneally administered each drug for 4 months, three times a week, at a dosage of 5 mg/kg (*n* = 11 per group). Vehicle-treated Tau^P301L^-BiFC mice and age-matched wild-type mice were used as control groups. Behavioral assessments were conducted as described below for one month from the end of drug administration. All mice were sacrificed at the age of 14 months for pathological analysis.

### Behavioral tests

#### Novel object recognition

The novel object recognition test was performed as described previously^41^. Briefly, for habituation, mice (*n* = 9 per group) were individually placed in the center of an open field arena (40 × 40 cm) and could freely explore for 15 min. The next day, in the training trial (familiarization phase), the mice could explore two identical objects in the open field for 10 min. In the testing trial (recognition phase) performed 24 h later, one familiar object was changed to a novel object that was different in color and shape. The mice could explore the objects for 10 min. The exploration time for the familiar (old) or the new (novel) object during the recognition phase was recorded using a Noldus EthoVision XT video tracking system. Memory was operationally defined by the recognition index calculated by dividing the time an animal spent exploring the novel object or old object by the total time spent exploring objects in the testing period. The exploration time was measured when the mouse pointed toward the object in the vicinity of the object.

#### Y-maze

Mice (*n* = 11 per group) were tested for spontaneous alternation behavior in a Y-shaped maze (60 cm in length of each arm, 20 cm in depth) using a standard protocol^42^. Spontaneous alternation for 8 min was calculated as the proportion of alternations. Alteration (%) was defined as consecutive entries in three different arms (ABCs) divided by the number of possible alterations (total arm entries minus 2)^43^.

#### Passive avoidance

The passive avoidance test was performed as described previously^44^ with the following modifications. Mice (*n* = 8 per group) were adapted in the passive avoidance chamber (Gemini) for 10 min and then returned to their home cages. The chamber is composed of a light compartment and a dark compartment separated by a connecting gate. The following day, the mice were placed in the light compartment, and the gate was opened after 30 s. When mice entered the dark compartment, the gate was closed, and an electrical foot shock (3 mA) was delivered for 2 s. The mice were left in the dark compartment for 30 s after the foot shock so that they associated the environment with the aversive stimulus. The mice were then returned to their home cages. The following day, mice were placed in the light compartment again, and the gate was opened after 30 s. The step-through latency, the time required for mice to enter the dark compartment, was measured up to 540 s.

### Preparation of brain tissue slices

Mice were perfused with 0.9% saline and fixed with PBS (pH 7.4) containing 4% paraformaldehyde (Sigma). Brains were extracted and fixed in PBS containing 4% paraformaldehyde at 4 °C for 48–72 h. For cryoprotection, the brains were transferred to PBS containing 30% sucrose solution and incubated at 4 °C until they sunk. For cryosectioning, the brains were embedded with O.C. T (Tissue-TEK) and cut serially using a cryostat (CM1860UV, Leica). Thirty-micron-thick tissue slices were maintained in PBS containing 0.05% sodium azide at 4 °C.

### Sudan Black B staining and BiFC fluorescence imaging

Brain tissue sections (*n* = 8 per group) were mounted onto slides. To reduce autofluorescence, brain tissue sections were stained with Sudan Black B solution (70% ethanol containing 0.05% Sudan Black B) for 10 min, washed three times with PBS containing 0.1% Triton X-100 (Sigma), and then washed with distilled water. For counterstaining of nuclei, brain tissues were stained with Hoechst (0.5 μg/mL) for 30 min. BiFC fluorescence (λ_ex_ = 460–490 nm and λ_em_ = 500–550 nm) images were acquired using Axio Scan. Z1 (ZEISS). For quantitative analysis, the mean BiFC fluorescence intensity was measured in the somatosensory cortex (layer V) and hippocampal CA1 from AP;−1.82. To normalize the background fluorescence between the brain samples, the habenular region was used as an internal control. Using ImageJ software (NIH), the region of interest was masked, and the fluorescence intensity value was calculated. Data are shown as the average and standard deviation of the mean fluorescence intensities of brain images from six to eight animals.

### Immunofluorescence analysis of brain tissue slices

For immunofluorescence image analysis, brain tissue slices (*n* = 6–8 per group) were stained with AT8 (pS202/T205) antibody (1:200, Invitrogen). The primary antibody was detected using Alexa Flour 633-conjugated anti-mouse antibody (1:500, Abcam). Fluorescence images (λ_ex_ = 620–640 nm and λ_em_ = 650–700 nm) were acquired using Axio Scan. Z1 (ZEISS).

### Preparation of RIPA-soluble and RIPA-insoluble brain lysates

Brains were weighed and suspended in RIPA lysis buffer (Sigma) containing protease and phosphatase inhibitor cocktails. Then, tissues were mechanically disrupted using a cordless mortar and pestle (Sigma) and incubated at 4 °C for 2 h with shaking. The homogenates were centrifuged at 20,000 × *g* at 4 °C for 20 min. The supernatants were collected as RIPA-soluble fractions and stored at −80 °C. To prepare RIPA-insoluble fractions, the remaining pellets were washed once with RIPA lysis buffer containing 1 M sucrose, resuspended in 2% SDS solution (1 mL per gram of tissue) and incubated at RT for 1 h. The mixtures were centrifuged at 20,000 × *g* for 1 min at RT. The supernatant was collected as RIPA-insoluble fractions and preserved at −80 °C.

### Human brain samples

Normal subject and AD human brain samples were neuropathologically examined and prepared according to procedures previously established by the Boston University Alzheimer’s Disease Research Center (BUADRC). Next-of-kin provided informed consent for participation and brain donation. Institutional review board approval for ethical permission was obtained through the BUADRC center. This study was approved for exemption by the Institutional Review Board of the Boston University School of Medicine because it only included tissues collected from postmortem subjects not classified as human subjects. The study was performed in accordance with institutional regulatory guidelines and principles of human subject protection in the Declaration of Helsinki. The sample information is listed in Supplementary Table 1.

### Statistics

All data in the quantitative analysis are presented as the mean ± S.D. or S.E.M. Unpaired *t tests* were performed when two groups were compared. One-way ANOVA or two-way ANOVA was performed when multiple groups were compared, depending on the number of independent variables. Statistical analysis was performed using GraphPad Prism 6 (GraphPad Software).

## Results

### Screening the FDA-approved & Passed Phase I Drug Library revealed levosimendan as a new anti-tau oligomerization agent

To identify a new drug candidate inhibiting tau aggregation, we screened 1018 compounds in the FDA-approved & Passed Phase I Drug Library on tau-BiFC cells. For tau-BiFC screening, tau-BiFC cells were plated in 384-well plates and cotreated with each library drug with forskolin (FK), which induces tau hyperphosphorylation through the activation of protein kinase A^45^ (Fig. 1a). MB and LMTM (3 μM) were used as positive controls. After 46 h, FK treatment induced tau aggregation, as shown by a 3.0 ± 0.4-fold increase in BiFC fluorescence intensity (Fig. 1a–d). FK treatment also induced cell cytotoxicity by decreasing the number of nuclei by 26.6 ± 0.2% (Fig. 1b, c, e). Among those tested, only one drug, levosimendan, suppressed FK-induced tau aggregation almost completely to the basal level without showing any cytotoxic effects (EC_50_ = 1.9 ± 0.1 μM, GI_50_ = 124.0 ± 0.3 μM) (Fig. 1f and Supplementary Fig. 1). More interestingly, FK-induced cell toxicity was rescued to the basal level by levosimendan treatment. In comparison, MB and LMTM decreased cell viability near the effective concentration (MB; EC_50_ = 2.8 ± 0.2 μM, GI_50_ = 6.2 ± 0.4 μM, and LMTM; EC_50_ = 2.2 ± 0.2 μM, GI_50_ = 6.4 ± 0.3 μM) (Fig. 1f and Supplementary Fig. 1).

Through Tau-BiFC cell screening, levosimendan was identified as an effective drug in preventing FK-induced tau aggregation and FK-induced cellular toxicity. However, FK activates not only tau kinases but also other cellular processes activated with the increased level of cAMP^46^. The inhibitory effect of levosimendan could be the result of regulating the upstream cAMP pathway rather than tau pathology. To scrutinize the drug effect on tau pathology, tauK18^P301L^ was used to activate tau aggregation. P301L mutation is a strong mutation that causes tau aggregation associated with frontotemporal dementia and parkinsonism linked to chromosome 17 (FTDP-17). Due to the self-aggregation propensity induced by P301L, purified TauK18^P301L^ proteins spontaneously assemble into oligomers (Supplementary Fig. 2a), which can initiate tau aggregation in tau-BiFC cells and primary neurons, acting as prion-like seeds^19^. When treated to tau-BiFC cells (Supplementary Fig. 2b), tauK18^P301L^ oligomers activated tau aggregation, as shown by a 2.9 ± 0.2-fold increase in BiFC fluorescence intensity. TauK18^P301L^ treatment also induced cell cytotoxicity by showing a 27.5 ± 0.3% decrease in the number of nuclei. Again, levosimendan almost completely prevented tauK18^P301L^-induced tau aggregation and cellular toxicity (EC_50_ = 2.2 ± 0.1 μM, GI_50_ = 101.3 ± 0.4 μM) (Fig. 1g and Supplementary Fig. 2c). MB and LMTM showed significant cell toxicity at effective concentrations (MB; EC_50_ = 3.1 ± 0.3 μM, GI_50_ = 7.1 ± 0.3 μM, and LMTM; EC_50_ = 2.7 ± 0.2 μM, GI_50_ = 6.6 ± 0.3 μM) (Fig. 1g and Supplementary Fig. 2c).

Next, flow cytometry analysis was conducted to investigate the drug effect at the single-cell level. Since MB and LMTM caused substantial cell death at effective concentrations, flow cytometry analysis was necessary to validate the drug effect on the remaining intact cells. For flow cytometry analysis, tau-BiFC cells were treated with the most effective concentration of each drug. Upon treatment with tauK18^P301L^, 65.4% of total cells were selected as an intact cell population in the forward and side scatter pulse areas (FSC-A and SSC-A) (Fig. 1h). In the case of MB and LMTM (5 μM), 13.7% and 14.5% of total cells were selected as an intact cell population. In comparison, 69.0% of levosimendan (15 μM)-treated cells were selected as intact cells. The selected populations were analyzed for BiFC fluorescence intensity. Tau-BiFC cells are a mixture of BiFC-null cells and BiFC-responding cells (Fig. 1i). Under basal conditions, BiFC-responding cells showed a low and widespread BiFC fluorescence profile (BiFC^OFF^ cells; median = 332 ± 110). Upon tauK18^P301L^ activation, the fluorescence intensities of BiFC-responding cells increased significantly, generating a distinctive population peak (BiFC^ON^ cells; median = 717 ± 62), while BiFC-null cells remained. The fluorescence intensity of BiFC^ON^ cells dropped significantly after levosimendan treatment (Levo; Median = 249 ± 59), demonstrating the effectiveness of levosimendan in inhibiting cellular tau aggregation. In comparison, MB or LMTM only slightly reduced tau-BiFC intensity (MB; Median = 648 ± 49, and LMTM; Median = 596 ± 57). High magnification images show the representative BiFC fluorescence images of each group (Fig. 1j). Upon treatment with tauK18^P301L^, intracellular tau aggregation increased significantly, showing a thread-like phenotype, and the increased BiFC fluorescence was suppressed almost completely by levosimendan treatment. As shown in the flow cytometry analysis, MB and LMTM were not effective in reducing intracellular tau aggregation.

### Levosimendan inhibits intracellular disulfide-linked tau oligomerization, while MB and LMTM increase disulfide-linked tau oligomers

**Fig. 2:**
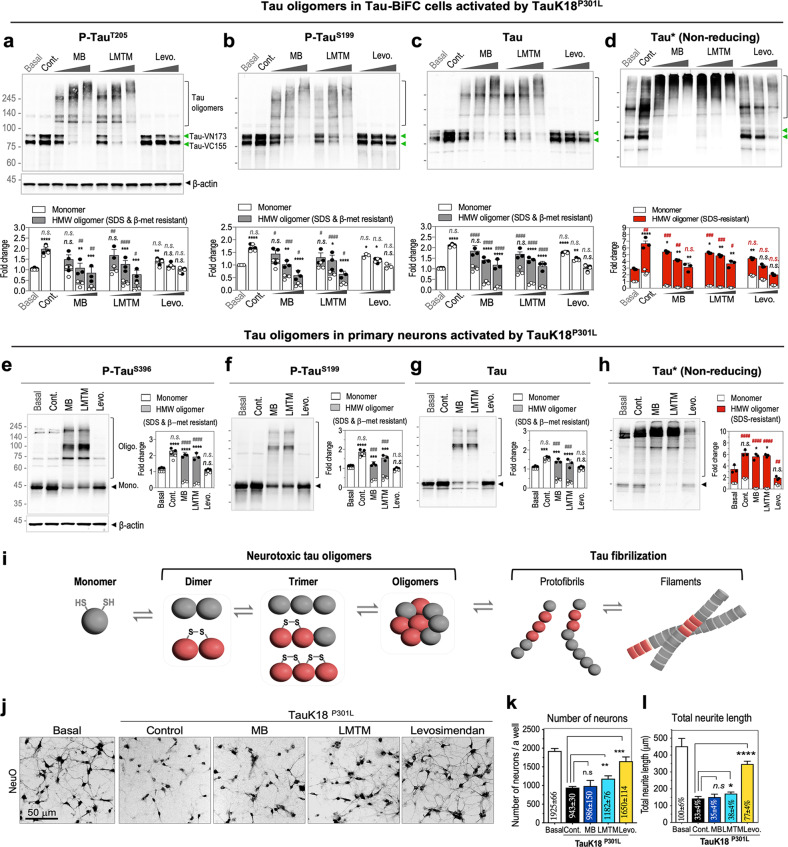
Levosimendan inhibits tau oligomerization in tau-BiFC and primary neuronal cells, while MB and LMTM increase tau oligomerization. **a**–**c** Immunoblot analysis of total and phospho-tau. For immunoblot analysis, tau-BiFC cells were treated with MB, LMTM (0.5, 1.5, 5 µM), or levosimendan (5, 15, 45 µM) in the presence of tauK18^P301L^. Green arrows indicate monomer bands of hTau-VN173 and hTau-VC155. Bands above 100 kDa indicate tau oligomers. Relative amounts of monomeric tau (white bars) and oligomeric tau (gray bars) were quantified by ImageJ. β-Actin was used as a loading control. All data were normalized to β-actin. **d** Immunoblot analysis of total tau under nonreducing conditions. Relative amounts of monomeric tau (white bars) and oligomeric tau (red bars) were quantified by ImageJ. **e**–**g** Immunoblot analysis of total and phospho-tau in the lysates of primary neurons. Relative amounts of monomeric and oligomeric tau were quantified by ImageJ. β-Actin was used as a loading control. All data were normalized to β-actin. **h** Immunoblot analysis of total tau under nonreducing conditions. **a**–**h** Data represent the mean ± S.D. of three independent experiments. Two-way ANOVA with Dunnett’s multiple-comparisons test was performed; *p < 0.05, **p < 0.01, ***p < 0.001, ****p < 0.0001, compared with the level of monomer in basal. ^*#*^*p* < 0.05, ^*##*^*p* < 0.01, ^*###*^*p* < 0.001, ^*####*^*p* < 0.0001, compared with the level of oligomers in basal. n.s. nonsignificant. **i** Schematic diagram of the tau aggregation process. **j** Representative images of primary hippocampal neurons treated with MB, LMTM, or levosimendan. On Day 10 in vitro, hippocampal neurons were treated with MB, LMTM (3 µM), or Levosimendan (10 µM) in the presence of TauK18^P301^. After 48 h, hippocampal neurons were stained with NeuO, a live-neuron selective probe, and imaged by using Operetta®. Scale bar, 50 µm. Quantification of the number of NeuO-stained neurons (**k**) and total neurite lengths (**l**). The number of neurons and total lengths of neurites were quantified by Harmony 3.1 software. Error bars represent the standard deviation of four replicate experiments. One-way ANOVA with Dunnett’s multiple-comparisons test was performed; *p < 0.05, **p < 0.01, ***p < 0.001, ****p < 0.0001, compared with the control.

Next, immunoblot analysis was performed to evaluate tau phosphorylation and total tau levels in tau-BiFC cells. To investigate the dose-dependency of each drug, tau-BiFC cells were treated with increasing concentrations of each drug in the presence of tauK18^P301L^ (MB and LMTM; 0.5, 1.5, 5 μM, and levosimendan; 5, 15, 45 μM). Upon activation by tauK18^P301L^, the levels of tau phosphorylation were increased by 1.9 ± 0.2-fold at T205 and 1.6 ± 0.1-fold at S199 compared to basal levels (Fig. 2a, b). The increased tau phosphorylation was reduced by levosimendan treatment. At the highest concentration (45 μM), the levels of tau phosphorylation were decreased almost to the basal level, showing 1.0 ± 0.1-fold at T205 and 0.9 ± 0.1-fold at S199 (Fig. 2a, b). Upon treatment with MB and LMTM, the level of tau monomers was dose-dependently decreased, but instead, high-molecular weight oligomers were observed. In the case of MB (5 μM), the level of pT205- or pS199-positive monomers was decreased to 0.1 ± 0.1- or 0.2 ± 0.1-fold, and the level of corresponding oligomers was increased to 0.7 ± 0.3- or 0.4 ± 0.2-fold, respectively, compared to that of basal. In the case of LMTM (5 μM), the level of pT205- or pS199-positive monomers was decreased 0.2 ± 0.2- or 0.3 ± 0.0-fold, and the level of oligomers was increased 0.6 ± 0.2- or 0.4 ± 0.1-fold, respectively. This result indicates that MB and LMTM increase unbreakable tau oligomers, which are resistant to SDS- and β-mercaptoethanol. Additionally, the size of oligomers was increased dose-dependently in MB- and LMTM-treated cell lysates.

Upon treatment with tauK18^P301L^, the amount of total tau protein was increased 2.0 ± 0.1-fold compared to the basal level (Fig. 2c). TauK18^P301L^-induced tau accumulation corresponds to a previous report showing that tau is metabolized at much slower rates when pathologically activated^47^. Levosimendan treatment effectively suppressed tauK18^P301L^-induced tau accumulation in tau-BiFC cells, maintaining the total tau level at 1.0 ± 0.2-fold, which is comparable to the basal level. Next, we investigated the formation of disulfide-dependent tau oligomers on nonreducing SDS‒PAGE gels. Substantial amounts of disulfide-linked tau oligomers were detected even under basal conditions, and the level of disulfide-linked tau oligomers increased 2.4 ± 0.4-fold upon treatment with tauK18^P301L^ (Fig. 2d). Levosimendan treatment decreased the amount of disulfide-linked tau oligomers, which was comparable to the basal level (0.8 ± 0.2-fold at 45 μM). Levosimendan also decreased disulfide-linked tau oligomers in forskolin-activated tau-BiFC cells (Supplementary Fig. 3). In contrast, MB and LMTM intensely increased the level of disulfide-linked tau oligomerization (MB; 1.6 ± 0.3-fold and LMTM; 1.9 ± 0.4-fold at 5 μM), presenting highly enriched bands above 245 kDa (Fig. 2d). Nonreducing gel analysis indicates that levosimendan is effective in inhibiting tau-BiFC responses through the suppression of disulfide-linked tau oligomerization. In comparison, MB and LMTM did not inhibit tau-BiFC responses and promoted the formation of structurally stable tau oligomers that are resistant to SDS and reducing agents. MB and LMTM also increased disulfide-linked tau oligomerization in forskolin-activated tau-BiFC cells (Supplementary Fig. 3). However, careful validation is necessary since it is possible that MB and LMTM might act on the BiFC complementation process instead of tau oligomerization.

Next, we evaluated the drug effect on tau oligomerization in primary neuron culture. Primary hippocampal neurons were isolated from Day 18 rat embryos. After 10 days of in vitro culture, the hippocampal neurons were treated with MB, LMTM (3 μM) or levosimendan (10 μM) in the presence of tauK18^P301L^. For MB and LMTM, severe neuronal cell death was observed at higher concentrations over 10 μM, similar to the toxicity on tau-BiFC cells (data not shown). The neuronal cell lysates were subjected to immunoblot analysis under both reducing and nonreducing conditions. Upon treatment with tauK18^P301L^, tau phosphorylation increased 2.0 ± 0.3-fold at S396 and 1.7 ± 0.1-fold at S199, indicating that tau pathology was activated in primary neurons (Fig. 2e, f). Again, levosimendan treatment suppressed tauK18^P301L^-induced tau phosphorylation comparable to the basal level, showing 0.9 ± 0.1-fold at S396 and 0.9 ± 0.1-fold at S199. Additionally, in primary neurons, treatment with MB and LMTM increased the level of high-molecular-weight tau oligomers resistant to SDS- and β-mercaptoethanol while decreasing the level of monomers (Fig. 2e, f). Upon treatment with tauK18^P301L^, the level of total tau increased 1.5 ± 0.1-fold compared to the basal level, indicating that pathological tau species were accumulated in neurons (Fig. 2g). Levosimendan treatment also suppressed tau accumulation in neurons, maintaining the total tau level at 1.0 ± 0.1-fold that of the basal level.

Nonreducing SDS‒PAGE analysis also revealed that a substantial amount of tau exists as disulfide-linked tau oligomers in primary neurons (Fig. 2h). Under basal conditions, the level of disulfide-linked oligomers was 2.4 ± 0.7-fold higher than the level of monomers. Upon tauK18^P301L^ activation, the amount of disulfide-linked tau oligomers was increased by 1.8 ± 0.3-fold compared to that of basal levels. Levosimendan efficiently suppressed the formation of disulfide-linked oligomers, decreasing the level of oligomers to 0.5 ± 0.1-fold, which is even lower than that of basal. For MB and LMTM, the level of oligomers was increased 2.3 ± 0.5- and 2.4 ± 0.3-fold, respectively, and monomers almost disappeared (Fig. 2h). Our results clearly indicated that while MB and LMTM increase the formation of tau oligomers in primary neurons, levosimendan prevented tau phosphorylation, inhibiting disulfide-linked tau oligomerization.

Nonreducing SDS‒PAGE analysis revealed that a significant amount of tau exists as disulfide-linked oligomers upon the activation of tau pathology. Full-length human tau contains two cysteine residues (C291 and C322) that can form intra- and intermolecular disulfide bonds^48^. While intramolecular disulfide bonds lead to the formation of compact monomers that cannot form extended structures, intermolecular disulfide bonds produce covalently linked oligomers. The disulfide-linked oligomers serve as “nuclei” for further tau aggregation^24^ (Fig. 2i). Tau aggregation may occur in the absence of disulfide bond formation, but disulfide-linked tau oligomers could facilitate tau aggregation, serving as a structural seed for tau aggregation. To date, accumulating evidence has reported the presence of SDS-resistant tau oligomers in the brains of AD patients^49–52^. In previous studies, SDS-resistant tau oligomers were even referred to as cross-linked multimers; however, the type of tau cross-link has not been identified^49^. To demonstrate the presence of disulfide-linked tau oligomers in the patient’s brain, we analyzed human brain lysates of AD and age-matched controls (Non-AD) obtained from the Boston University Alzheimer’s Disease Research Center (BUADRC) (Supplementary Fig. 4). The brain lysates were analyzed by reducing and nonreducing SDS‒PAGE analysis. Under nonreducing conditions, significant amounts of SDS-stable and high molecular weight tau oligomers were detected in both AD patients and normal subjects. This result correlates with the results of primary neuron culture, indicating that tau disulfide-bond formation frequently occurs in mouse and human neurons. The amount of disulfide-linked tau oligomers was 1.8-fold higher in the brains of AD patients than in those of age-matched controls (Supplementary Table 1). The disulfide-linked oligomers were positive for the AT8 antibody, indicating that pathological tau oligomers are disulfide-linked oligomers. Our results indicate that treatment with MB and LMTM increased the level of disulfide-linked tau oligomers and that levosimendan prevented the formation of disulfide-linked tau oligomers.

Next, we evaluated the effects of tau oligomers on neuronal integrity. At DIV10, hippocampal neurons were treated with MB, LMTM (3 μM) or levosimendan (10 μM) followed by tauK18^P301L^. After 48 h of incubation, neurons were stained with NeuO (NeuroFluor^TM^ NeuO), which is a live-neuron selective probe^40^ (Fig. 2j). The number of NeuO-stained neurons and total lengths of neurites were quantified by Harmony 3.1 software. Error bars represent the standard deviation of four replicate experiments (Fig. 2k, l). Under basal conditions, 1925 ± 66 live neurons were stained by NeuO. Upon treatment with tauK18^P301L^, the number of live neurons was decreased to 943 ± 30, indicating tau-induced neuronal cell toxicity (Fig. 2k). Compared to MB and LMTM, levosimendan suppressed tau-induced neuronal toxicity effectively, as the number of live neurons was significantly increased (levosimendan; 1650 ± 114 neurons, MB; 986 ± 150 neurons, LMTM; 1182 ± 76 neurons). In addition, significant tauK18^P301L^-induced neuronal degeneration was observed, as 142.8 ± 17.8 μm shortened neurites were present compared to basal neurites (*p* < 0.0001) (Fig. 2l). Levosimendan inhibited tauK18^P301L^-induced neuronal degeneration by recovering neurite length to 346.8 ± 20.7 μm. In contrast, MB did not show a significant effect on neuronal integrity, and LMTM slightly protected neuronal degeneration by showing a neurite length of 151.1 ± 24.4 μm. This result clearly indicates that levosimendan prevented tauK18^P301L^-induced toxicity not only in tau-BiFC cells but also in primary neurons by inhibiting the formation of disulfide-dependent tau oligomers.

In addition, immunoblot analysis against three major tau kinases was conducted to investigate whether MB and LMTM treatment significantly increased ERK1/2 phosphorylation in HEK293 and primary neurons (Supplementary Fig. 5). This result corresponds with increased tau phosphorylation.

### Levosimendan not only prevents tau oligomerization but also disaggregates filamentous tau

**Fig. 3:**
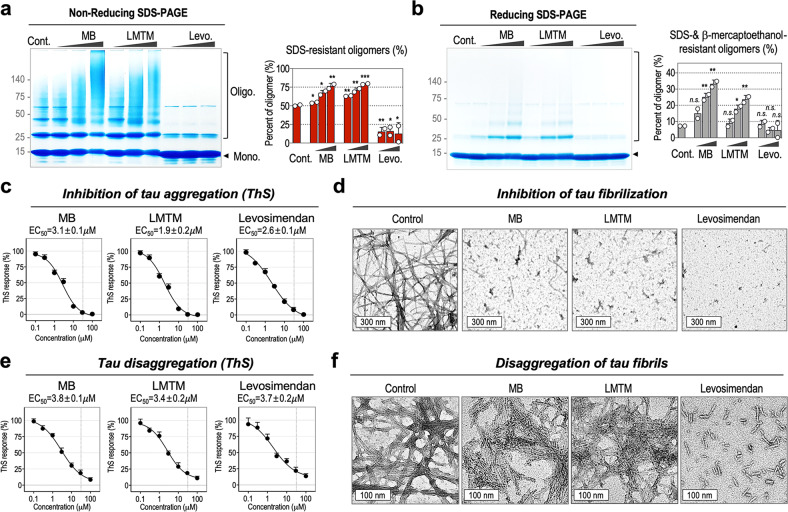
Levosimendan inhibits disulfide-dependent tau oligomerization in vitro. Nonreducing (**a**) and reducing (**b**) SDS‒PAGE analysis of tau oligomerization in vitro. Tau preaggregates were incubated with MB, LMTM, or levosimendan (10, 30, 100 µM) for 5 h at RT, and tau proteins were separated on an SDS‒PAGE gel (4–20%) under reducing or nonreducing conditions. Tau oligomers on SDS‒PAGE gels were visualized with Coomassie blue staining. The black arrow indicates the tau monomer (14 kDa). Bands above 25 kDa indicate tau oligomers. Relative amounts of tau monomers and oligomers were quantified by ImageJ. The percentages of SDS-resistant tau oligomers are indicated as red bars (**a**), and the percentages of SDS- and β-mercaptoethanol-resistant oligomers are indicated as gray bars (**b**). Data represent the mean ± S.D. of two independent experiments. n.s. nonsignificant, *p < 0.05, **p < 0.01, ***p < 0.001, compared with control. **c** EC_50_ curves of MB, LMTM, or levosimendan in inhibiting tau aggregation. TauK18 aggregation was induced by heparin and DTT in the presence of each drug for 5 days. The level of β-sheet aggregation was determined with thioflavin S (ThS). Data represent the mean ± S.D. of three independent experiments. Prism’s nonlinear regression analysis was used to measure the EC_50_ values. **d** Representative transmission electron microscopy images indicating the inhibitory effect of MB, LMTM, and levosimendan at 30 µM. Scale bar, 300 nm. **e** EC_50_ curves of MB, LMTM, or levosimendan on disaggregating tau filaments. For the disaggregation assay, tau aggregates were incubated with each drug at various concentrations for 4 days. The level of β-sheet aggregation was determined with thioflavin S (ThS). Prism’s nonlinear regression analysis was used to measure the EC_50_ values. Data represent the mean ± S.D. of three independent experiments. **f** Representative images of transmission electron microscopy indicating the drug’s effects on tau disaggregation at 30 µM. Scale bar, 100 nm.

Next, we validated the direct effect of each drug on tau oligomerization and tau fibrilization under in vitro conditions using a purified tauK18 fragment. For tau oligomerization analysis, tau preaggregates were incubated with each drug at various concentrations (10, 30, and 100 μM) for 5 h. Then, tau oligomers in the mixtures were separated by reducing and nonreducing SDS‒PAGE (Fig. 3a, b). In the DMSO-treated control, 50 ± 1.1% of total tau existed as disulfide-linked oligomers, which formed spontaneously in phosphate-buffered saline (pH 7.4). Upon levosimendan treatment, the levels of disulfide-linked tau oligomers were decreased to 15.0 ± 6.0% at 10 μM, 15.7 ± 7.8% at 30 μM, and 12.3 ± 15.0% at 100 μM, increasing the level of monomers. This result indicates that levosimendan directly inhibits disulfide-linked tau oligomerization. For MB and LMTM, at 100 μM, the level of disulfide-linked tau oligomers was increased to 77.6 ± 2.0% and 79.2 ± 0.8%, respectively. MB- and LMTM-induced tau oligomers are not easily dissociable into monomers even under reducing conditions, showing noticeable bands of dimers and trimers resistant to SDS- and β-mercaptoethanol on a reducing SDS‒PAGE gel (Fig. 3b). An in vitro tau oligomerization assay indicated that levosimendan prevents disulfide-linked tau oligomerization by direct interaction with tau protein, while MB and LMTM increase disulfide-linked tau oligomerization.

Then, we investigated the inhibitory effect on tau aggregation. For the tau aggregation assay, tau aggregation was induced by the treatment of heparin to purified tauK18 in the presence of each drug at various concentrations for 5 days. The formation of β-sheet aggregates was evaluated with a thioflavin S (ThS) assay. All drugs inhibited the formation of ThS-positive aggregates at micromolar concentrations (MB; EC_50_ = 3.1±0.1 μM, LMTM; EC_50_ = 1.9 ± 0.2 μM, and levosimendan; EC_50_ = 2.6 ± 0.1 μM) (Fig. 3c). Then, the structures of tau aggregates were evaluated under transmission electron microscopy (TEM) (Fig. 3d). The TEM image of the DMSO control shows the formation of long and straight filaments of tau. In comparison, tau filaments were not observed in the levosimendan-treated mixture, indicating its effectiveness in inhibiting tau aggregation. For MB and LMTM, a few thread-like filaments were observed. We also investigated the ability of the drugs to reverse tau aggregation. For the disaggregation assay, tau aggregates were treated with each drug at various concentrations for 4 days. The ThS assay indicated that all three drugs were effective in reducing ThS-positive tau aggregates (MB; EC_50_ = 3.8 ± 0.1 μM, LMTM; EC_50_ = 3.4 ± 0.2 μM, and levosimendan; EC_50_ = 3.7 ± 0.2 μM) (Fig. 3e). However, the ThS results of MB and LMTM conflicted, and the TEM images showed thick bundles of tau filaments in the MB- and LMTM-treated mixture (Fig. 3f). This result corresponds with a previous study showing that upon treatment of tau filaments, MB did not reduce the tau filaments shown under an electron microscope^53^. Our results imply that treatment with MB or LMTM prevents the interaction between ThS and tau filaments, generating ThS-negative tau filaments. For levosimendan, the ThS response correlated with the high-resolution TEM image, which shows many short tau fragments. The lengths of tau filaments range from 8.8 to 139.3 nm (*n* = 403). The in vitro tau aggregation and disaggregation assay shows the effectiveness of levosimendan as an anti-tau oligomerization agent, which not only inhibits tau aggregation but also disassembles tau filaments.

To further confirm the effectiveness of levosimendan on tau disassembly, levosimendan was administered at diverse time points during tau aggregation processes in vitro and in tau-BiFC cells. For the in vitro assay, tauK18 aggregation was induced by heparin, and the level of tau aggregation was monitored with ThS over 5 days. Levosimendan was added to an aliquot of the aggregation mixture at 0, 25, 44, and 75 h after heparin activation (Supplementary Fig. 6a). The ThS-response curve shows that tau aggregation followed a nucleation–elongation mechanism. Levosimendan treatment, regardless of the administration time, decreased ThS-positive tau aggregates (Supplementary Fig. 6b). For the tau-BiFC assay, tau pathology was activated by treatment with tauK18^P301L^, and tau-BiFC fluorescence intensities were monitored over three days. Tau-BiFC cells were treated with levosimendan at 1, 12, 24, and 36 h after tauK18^P301L^ activation (Supplementary Fig. 6c). Again, levosimendan treatment, decreased tau-BiFC fluorescence responses, regardless of the administration time, indicating its effectiveness as an anti-tau oligomerization agent (Supplementary Fig. 6d, e).

### Levosimendan inhibits disulfide-crosslinked tau oligomerization in vitro

**Fig. 4:**
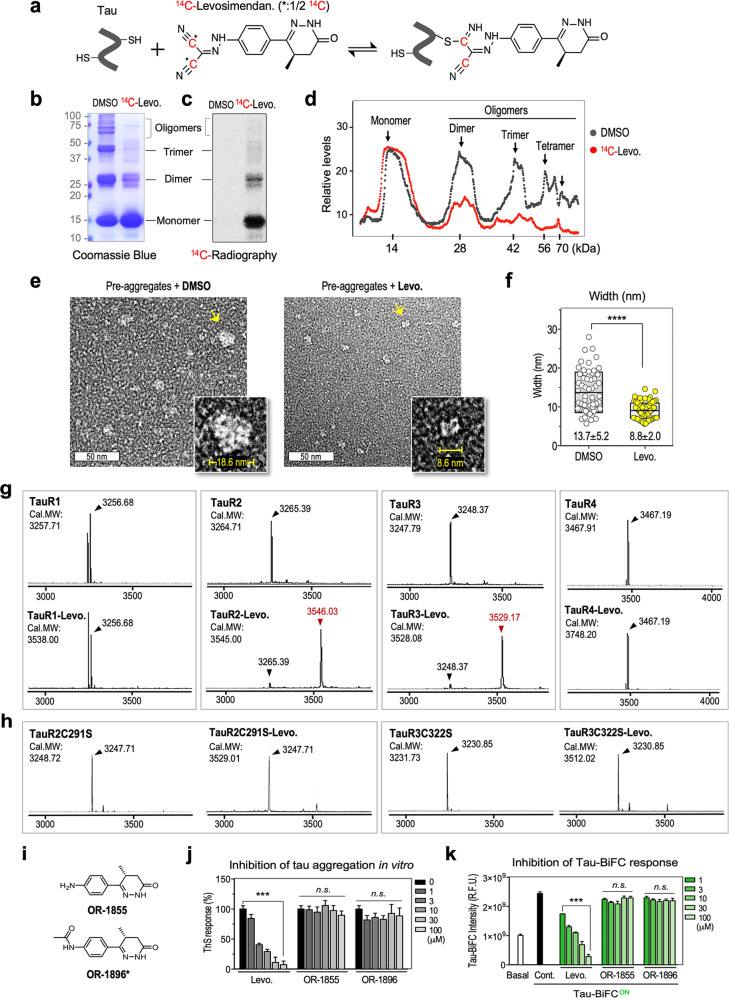
Levosimendan inhibits disulfide-linked tau oligomerization by capping tau cysteine residues. **a**–**c** Schematic diagram showing thioimidate bond formation between ^14^C-levosimendan and tau cysteine (**a**). TauK18 preaggregates were incubated with DMSO control or ^14^C-levosimendan for 2 h at RT. The mixtures were separated on a nonreducing SDS‒PAGE gel (15%) for further analysis. Coomassie blue staining indicates disulfide-linked tau oligomers on the SDS‒PAGE gel (**b**). The SDS‒PAGE gel was transferred onto a PVDF membrane, and ^14^C-labeled tau proteins were visualized by ^14^C autoradiograph (**c**). **d** The relative levels of tau monomer and oligomers on the Coomassie Blue-stained SDS‒PAGE gel. Two independent experiments were performed. **e** Representative transmission electron microscopy images indicating the disassembly of tau oligomers. A representative tau particle from each group is indicated with a yellow arrow, and the magnified image indicates the particle’s width. Scale bar, 50 nm. **f** Quantification of the widths of tau particles on TEM images. The width of tau particles was measured by ImageJ. Unpaired *t test* was performed; ****p < 0.0001. MALDI-TOF analysis of tau repeat domains (R1-R4) (**g**), R2 containing a C291S mutation and R3 containing a C322S mutation (**h**) in the absence and presence of levosimendan. Each repeat domain was incubated with levosimendan for 36 h at RT (molar ratio 1:10 of a repeat domain: levosimendan). **i** Structures of OR-1855 and OR-1896, which are not thiol-reactive. **j** Evaluation of the inhibitory effect of OR-1855 and OR-1896 on tau aggregation in vitro by ThS. Data represent the mean ± S.D. of three repeated experiments. One-way ANOVA with Dunnett’s multiple-comparisons test was performed; ***p < 0.001, n.s. nonsignificant. **k** Evaluation of the inhibitory effect of OR-1855 and OR-1896 on tau-BiFC aggregation. Data represent the mean ± S.D. of three repeated experiments. One-way ANOVA with Dunnett’s multiple-comparisons test was performed; ***p < 0.001, n.s. nonsignificant.

Next, we investigated the molecular mechanism by which levosimendan inhibits oligomerization. We hypothesized that levosimendan inhibits tau-disulfide bond formation by blocking tau cysteine residues since its nitrile group can form a thioimidate bond with cysteine. To verify its covalent modification to tau, one of two nitrile groups of levosimendan was labeled with a ^14^C-radioisotope (Fig. 4a and Supplementary Materials). ^14^C-levosimendan was administered to tau preaggregates that contained disulfide-linked tau oligomers for 2 h. Then, disulfide-linked tau oligomers were separated on a nonreducing SDS‒PAGE gel (Fig. 4b, c). ^14^C-radiography showed tau monomers and dimers labeled with ^14^C-levosimendan, indicating its covalent attachment to tau (Fig. 4c). Then, the relative intensities of the Coomassie blue-stained bands of the monomer, dimer, trimer, and tetramer were compared (Fig. 4c, d). Corresponding to Fig. 3e, treatment with ^14^C-levosimendan disassembled disulfide-linked tau oligomers, increased the level of monomers as levosimendan did (Fig. 4c, d). In addition, high-resolution TEM images were acquired to validate the size of tau oligomers in the DMSO-treated control and levosimendan-treated mixture. Heterogeneous tau particles with diameters of 13.7 ± 5.2 nm were observed in the DMSO-treated control (*n* = 60) (Fig. 4e, f). The diameters of tauK18 oligomers match those in a previous report showing that tauK18 formed spherical tau oligomers with diameters of 10–20 nm^54^. In comparison, comparably small tau particles with diameters of 8.8 ± 2.0 nm were observed in the levosimendan-treated mixture (*n* = 69), supporting the disassembly of tau oligomers by levosimendan.

To validate levosimendan binding to tau cysteine residues, each repeat domain (R1-R4) of the microtubule binding region was synthesized and incubated with levosimendan. MALDI-mass spectrometry indicated that levosimendan covalently attached to the R2 and R3 domains, which each contained a cysteine residue (Fig. 4g). When the cysteine was mutated to serine, levosimendan did not bind to the R2 or R3 domain, supporting that levosimendan binds to tau cysteines (Fig. 4h). In addition, to evaluate the reversibility of levosimendan, we performed NMR analysis of levosimendan with *N*-acetyl-L-cysteine methyl ester (Supplementary Fig. 7). *N*-acetyl-L-cysteine methyl ester serves as a simplified cysteine mimetic and is frequently used in thiol-addition reaction studies, including NMR studies^55^. When levosimendan was incubated with *N*-acetyl-L-cysteine methyl ester, the formation of the levosimendan-cysteine conjugate was completed within 2 h. To evaluate the reversibility of the reaction, the reaction mixture was diluted ten times. Upon dilution, cysteine dissociation proceeded slowly, and levosimendan was almost completely restored after 5 days, indicating that levosimendan reversibly binds to the cysteine-thiol. These NMR spectrum data indicated that levosimendan could form a reversible covalent bond with a cysteine residue.

Next, we confirmed the importance of the nitrile moiety of levosimendan in inhibiting tau aggregation by using its metabolites. In humans, 5% of levosimendan is metabolized to OR-1855 and OR-1896, in which the nitrile moiety is absent^56^ (Fig. 4i). In particular, OR-1896 is a pharmacologically active metabolite of levosimendan, acting as a powerful inodilator^57^. The metabolite’s effects on tau aggregation were evaluated in vitro and in tau-BiFC cells. For the in vitro assay, tau aggregation was induced by heparin in the presence of each metabolite for 5 days. ThS responses indicated that OR-1855 and OR-1896 did not inhibit tau aggregation in vitro, while levosimendan showed strong anti-tau aggregation activity (Fig. 4j). For the tau-BiFC assay, tau-BiFC cells were treated with each metabolite for 48 h in the presence of tauK18^P301L^. Again, even at 100 μM, OR-1855 and OR-1896 did not inhibit the tau-BiFC fluorescence increase (Fig. 4k). This result indicated that the nitrile group is critical in inhibiting tau aggregation through modifying tau cysteine residues. Interestingly, OR-1896, which is a pharmacologically active metabolite acting as an inodilator, did not show any effect on tau. This result strongly suggests that the anti-tau activity of levosimendan is a new mode of action different from its known function as an inodilator.

### Levosimendan rescues cognitive deficits and tau pathology in Tau^P301L^-BiFC mice

**Fig. 5:**
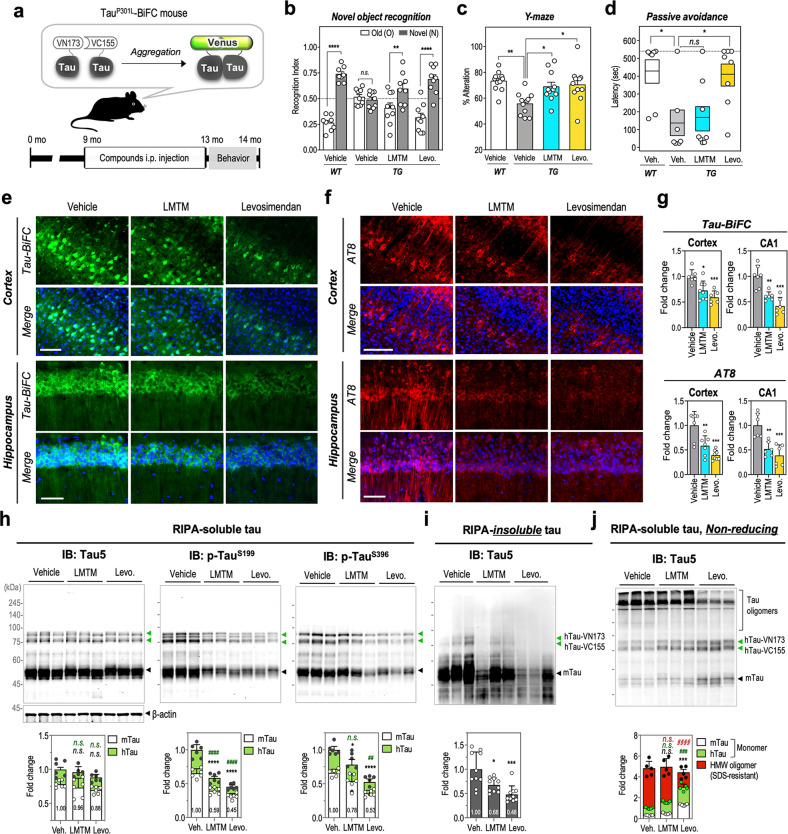
Levosimendan suppresses tau pathology and cognitive decline in aged Tau^P301L^-BiFC mice. **a** Diagram of the Tau^P301L^-BiFC mouse model and experimental design for administration of LMTM and levosimendan. Tau^P301L^-BiFC mice express a human tau mutant (P301L) fused with two nonfluorescent compartments of the Venus protein. Tau^P301L^-BiFC mice intraperitoneally received LMTM or levosimendan (5 mg/kg) from 9 months of age for 4 months, three times a week (*n* = 11). **b** Recognition index (RI) in a novel object recognition test (*n* = 9). RI was defined as the ratio between the times spent exploring the familiar object (Old) or novel object (Novel) divided by total exploratory time. An RI close to 0.5 (dashed line) indicates that the mice could not discriminate between novel and familiar objects. Two-way ANOVA with Sidak’s multiple-comparisons test was performed; **p < 0.01, ****p < 0.0001. **c** Percent alteration in the *y*-maze test (*n* = 11). A spontaneous alteration was calculated by dividing the number of mice that entered different arms of the maze in each of 3 consecutive arm entries by the total number of arm entries. One-way ANOVA with Tukey’s multiple-comparisons test was performed; *p < 0.05, **p < 0.01. **d** Latency to enter the dark compartment during the retention test of the passive avoidance test (*n* = 8). During the training test, each mouse received a foot shock (3 mA, 2 s) after entering the dark compartment. The retention test was performed 24 h after training. One-way ANOVA with Tukey’s multiple-comparisons test was performed; *p < 0.05. **e**, **f** Representative images of BiFC fluorescence (**e**) and AT8 immunofluorescence (F) in the cortex and hippocampus of Tau^P301L^-BiFC mice. Scale bar, 50 μm. **g** Quantification of the fluorescence intensities of tau-BiFC and AT8 immunofluorescence (*n* = 6–8). The mean fluorescence intensities were measured in layer V of the somatosensory cortex and CA1 of the hippocampus. The values were normalized to those of the vehicle-treated group. One-way ANOVA with Tukey’s multiple-comparisons test was performed; *p < 0.05, **p < 0.01, ***p < 0.001. **h** Immunoblot analysis of total tau and phosphorylated tau with Tau5, anti-p-Tau^S199^, and anti-Tau^S396^ antibodies in RIPA-soluble fractions of Tau^P301L^-BiFC brain lysates (*n* = 6). Green arrows indicate hTau-VN173 and hTau-VC155. Black arrows indicate endogenous murine tau. Relative amounts of human tau (hTau, green bars), murine tau (mTau, white bars) monomers, and total tau (gray bars) were quantified by ImageJ. β-Actin was used as a loading control. All data were normalized to β-actin. **i** Immunoblot analysis of total tau in RIPA-insoluble fractions. Total tau (gray bars) was quantified by ImageJ. **j** Immunoblot analysis of soluble tau oligomers in brain lysates. To evaluate disulfide-linked tau oligomers, RIPA-soluble brain lysates were separated on a nonreducing SDS‒PAGE gel (vehicle, LMTM, levosimendan; *n* = 10). Relative amounts of hTau (green bars), mTau (white bars) monomers, and oligomers (red bars) were quantified by ImageJ. **h**–**j** To indicate the significance of the results, two-way ANOVA with Tukey’s multiple-comparisons test was performed; *p < 0.05, ***p < 0.001, ****p < *0.0001*, compared with mTau of the vehicle-treated; ^*##*^*p* < 0.01, ^*###*^*p* < 0.001, ^*####*^*p* < 0.0001, compared with hTau of the vehicle-treated; ^***§§§§***^*p* < 0.0001, compared with an oligomer of the vehicle-treated; n.s. nonsignificant.

Next, we evaluated the in vivo efficacy of levosimendan in Tau^P301L^-BiFC mice^33^. The Tau^P301L^-BiFC mouse model expresses human mutant (P301L) tau labeled by BiFC compartments (Fig. 5a). In the brains of Tau^P301L^-BiFC mice, tau oligomerization occurs at 3 months, and neuronal degeneration occurs at 9 months, leading to cognitive deficits at 12 months of age. To evaluate the therapeutic effect of levosimendan in preventing neuronal degeneration and cognitive impairment, Tau^P301L^-BiFC mice received intraperitoneal administration of levosimendan or LMTM (5 mg/kg, three times per week) from 9 months to 12 months (Fig. 5a). After 4 months of drug administration, mice were subjected to behavioral tests to monitor cognitive function: the novel objective test (NOR), Y-maze test, and passive avoidance test. For comparison, age-matched wild-type (WT) littermates were also subjected to the tests. In the novel objective recognition test, cognitive abilities were determined by the recognition index (RI) for a novel object. Vehicle-treated transgenic (TG) mice showed a significant decrease in the recognition index, with an RI value of 0.49 (*p* < 0.0001, compared to WT*)*, while WT mice exhibited an RI value of 0.74 for a novel object. Levosimendan treatment significantly improved recognition memory performance with an RI value of 0.69 (*p* < 0.0001). LMTM treatment also attenuated the recognition deficit of TG mice by showing an RI value of 0.59 (*p* < 0.01) (Fig. 5b). In the Y-maze test, cognitive ability was determined by the percent alternation. Each levosimendan- and LMTM-treated group showed a significant increase in the alternation (70.4 ± 4.2%, *p* < 0.05 and 68.9 ± 3.5%, *p* < 0.05 compared to vehicle-treated, respectively) compared with the vehicle-treated group (52.8 ± 2.6%, *p* < 0.01 compared to WT) (Fig. 5c). Furthermore, a passive avoidance test was performed to assess emotion-associated learning ability. Learning abilities were determined by the latency of entering the dark compartment, in which the mice received an electrical shock a day before. Most mice in the vehicle-treated group entered the dark chamber without hesitation (*p* < 0.05), indicating that their memory of fear was impaired. Levosimendan-treated mice exhibited improved memory, showing delayed latency or not entering the chamber compared with the vehicle-treated TG controls (*p* < 0.05) (Fig. 5d). Interestingly, the LMTM-treated group did not show significant improvement similar to vehicle-treated TG mice. All these results indicate that the administration of levosimendan ameliorates tauopathy-induced memory deficits in aged, symptomatic tau TG mice.

Next, brains were extracted from mice, and brain tissue sections were prepared to evaluate tau pathology. Tau-BiFC fluorescence images indicate the level of tau assembly, including tau oligomers and aggregates, and AT8 immunostaining indicates the level of tau phosphorylation that is associated with tau aggregation at a late state. Tau-BiFC intensities decreased in both the LMTM- and levosimendan-treated groups (Fig. 5e, g). In the levosimendan-treated group, tau-BiFC intensities decreased to 0.6 ± 01-fold in the cortex and 0.4 ± 0.2-fold in the hippocampus compared to those of the vehicle-treated group. In the LMTM-treated group, tau-BiFC intensities decreased 0.7 ± 0.2-fold in the cortex and 0.6 ± 0.1-fold in the hippocampus. AT8 immunoreactivity also decreased in both the LMTM- and levosimendan-treated groups (Fig. 5f, g). In the levosimendan-treated group, AT8 immunoreactivity decreased 0.4 ± 0.1-fold in the cortex and 0.4 ± 0.2-fold in the hippocampus compared to the vehicle-treated group. In the LMTM-treated group, AT8 immunoreactivity decreased 0.6 ± 0.2-fold in the cortex and 0.5 ± 0.2-fold in the hippocampus.

Next, a tau immunoblot assay was performed to evaluate the effects of the drugs on tau oligomerization and aggregation using RIPA-soluble and RIPA-insoluble brain lysates. On the tau immunoblots, mouse tau is indicated with a black arrow (50 kDa), and human tau-BiFC compartments are indicated with two green arrows (76 and 85 kDa). β-Actin was used as an immunoblot loading control. In RIPA soluble brain lysates, tau phosphorylation levels were significantly decreased in the LMTM- and levosimendan-treated groups (Fig. 5h). In the levosimendan-treated group, the levels of tau phosphorylation decreased 0.5 ± 0.0-fold at S199 and 0.5 ± 0.1-fold at S396 compared to those in the vehicle-treated group. In the LMTM-treated group, the levels of tau phosphorylation decreased 0.6 ± 0.1-fold at S199 and 0.8 ± 0.2-fold at S396. In contrast to the previous results showing increased levels of SDS- and β-mercaptoethanol-resistant tau oligomers, the LMTM-treated group did not show any high-molecular-weight oligomers in the brain lysates (Fig. 5h). Additionally, the level of total tau decreased slightly in the levosimendan-treated group, although the decrease was not statistically significant. Corresponding to the AT8 immunofluorescence images in Fig. 5f, the amount of insoluble tau decreased 0.5 ± 0.2-fold in levosimendan-treated mice (Fig. 5i). In the case of LMTM, the amount of insoluble tau decreased 0.7 ± 0.1-fold. Collectively, our results indicated that levosimendan is a new drug candidate targeting tau oligomerization, suppressing tau phosphorylation and aggregation in Tau^P301L^-BiFC mice. Corresponding to the AT8 immunofluorescence images in Fig. 5f, the amount of insoluble tau decreased 0.5 ± 0.2-fold in levosimendan-treated mice (Fig. 5i). In the case of LMTM, the amount of insoluble tau decreased 0.7 ± 0.1-fold.

Next, the level of disulfide-linked tau oligomers was evaluated by nonreducing SDS‒PAGE analysis. Similar to the results of primary neuron cultures, disulfide-linked tau oligomers were observed in the brain lysates of wild-type mice (Supplementary Fig. 8). In Tau^P301L^-BiFC mice, most human and murine tau exists in the form of disulfide-linked oligomers (> 245 kDa) (Fig. 5j and Supplementary Fig. 8). Levosimendan treatment significantly decreased the high-molecular-weight oligomers and increased soluble monomeric tau levels. This result is consistent with previous data showing that levosimendan inhibits the formation of disulfide-cross linkage between tau. In contrast, LMTM treatment did not increase or decrease the level of disulfide-linked tau oligomers in the brain (Fig. 5j). The LMTM effect in the animal was contrasted with the effect when LMTM interacted with tau directly in test tubes. This result implies that when intraperitoneally administered to mice, LMTM might not directly interact with tau, as shown in vitro (Fig. 3a, b). The anti-tau activity of LMTM shown in the brains of Tau^P301L^-BiFC mice could result from methods that indirectly regulate the tau pathway, such as decreasing tau phosphorylation or increasing autophagic clearance of aggregated proteins^7,58^. Collectively, our results indicated that levosimendan is a new drug candidate targeting tau oligomerization, suppressing tau phosphorylation and aggregation in Tau^P301L^-BiFC mice.

## Discussion

Levosimendan was identified 20 years ago and has been used to treat patients with acute heart failure^59^. As an inodilator, levosimendan possesses both positive inotropic and vasodilator actions; (i) as a calcium sensitizer, it enhances the sensitivity of contractile proteins to calcium through covalent binding to troponin C^60^. (ii) As a vasodilator, levosimendan inhibits phosphodiesterase III and opens ATP-dependent K + channels in smooth muscle cells, leading to arteriolar and venous dilation^61^. In addition to the heart, ATP-dependent K + channels are present in a number of tissues, including the brain^62^. In 2010, Roehl et al. reported the neuroprotective effects of levosimendan in an in vitro model of traumatic brain injury, which might be associated with the activation of neuronal ATP-dependent K + channels^63,64^. There is also evidence indicating that the neuroprotective effect of levosimendan is associated with its action as a vasodilator. In 2015, Levijoki et al. reported that orally administered levosimendan increased the blood volume of the cerebral vessels, reducing mortality and morbidity in rat models of primary and secondary stroke^65^. In 2015, Matti Kivikko et al. reported a pilot study showing that low-dose oral administration of levosimendan increased cerebral blood flow velocities in patients with an earlier ischemic cerebrovascular event, supporting the vasodilatory effect in the brain circulation^66^.

Here, our study shows a new molecular mechanism of levosimendan as an anti-tau agent. By modifying tau cysteine residues, levosimendan inhibits disulfide-linked tau oligomerization. In contrast to LMTM, which showed conflicting effects on tau, levosimendan exhibited a consistent inhibitory effect on tau oligomerization in vitro, in cells, and in the brains of mice, suppressing tau aggregation. In tau-BiFC mice, 4 months of levosimendan administration not only suppressed tau pathology but also prevented memory deficits in aged Tau^P301L^-BiFC mice. In 2019, Rababa’h et al. reported that levosimendan could prevent memory impairment induced by a streptozotocin-induced diabetic model in rats^67^. In their study, streptozotocin-induced hyperglycemia increased oxidative stress in the hippocampus, causing memory impairment, and levosimendan prevented glycemia-induced memory impairment. The authors speculated that the antioxidant activity of levosimendan might be the major mechanism of memory protection in a diabetic model. Neuronal cells are highly sensitive to oxidative stress, and accumulated oxidative stress is among the key mechanisms contributing to cognitive aging and multiple neurodegenerative disorders, including AD^68,69^. Oxidative stress generates free radical attack on neural cells, leading to neuronal cell death associated with protein misfolding, glial cell activation, mitochondrial dysfunction, and subsequent cellular apoptosis. Especially in protein-misfolding diseases, proteins modified by oxidative reactive species tend to form aggregates, and highly oxidized and cross-linked proteins cause the cellular clearance system to break down. Therefore, both the antioxidant and anti-tau properties of levosimendan could be possible mechanisms of neuroprotection that prevent memory impairment. To apply levosimendan as an AD therapeutic, safety issues should be clarified due to its action as an inodilator. There is a strong association between dementia and cardiovascular disease. Cardiovascular insufficiency impairs the function of diverse organs, including the brain, which can worsen pathology related to dementia^70,71^. As an example, phosphodiesterase III is known to be upregulated in the cerebral blood vessels of AD patients due to vascular amyloid burden, and phosphodiesterase III inhibitors have shown protective effects in an AD model^72,73^. In that case, the vasodilator action of levosimendan would be beneficial for improving the function of AD brains.

Drug repositioning is an attractive drug discovery strategy that develops new therapeutic value from existing drugs^74^. MB is among the representative cases of drug repositioning. In 1891, MB was first approved to treat malaria with its activity as a chloroquine sensitizer^75^. MB has been approved to treat clinical pain syndromes, psychotic disorders, cyanide poisoning and urinary tract infections^76^. In 2016, MB was approved to treat methemoglobinemia^77^. In addition to the known medical actions of MB, many studies have compiled a wide variety of biological activities of MB, which (i) inhibits the activity of monoamine oxidase A^78^, nitric oxide synthase^79^, and guanylate cyclase^80^, (ii) increases the release of neurotransmitters, such as serotonin and norepinephrine^81,82^, (iii) increases cholinergic transmission^83^, iv) inhibits GSK3β and microtubule-affinity regulating kinases^84,85^, and (v) promotes autophagic clearance of β-amyloid^86^ and tau^87^. The action of methylene blue on multiple targets in the brain justifies its symptom-relieving effects on Tau^P301L^-BiFC mice, improving learning and cognitive abilities and reducing tau phosphorylation and aggregates. However, our in vitro and cell-based data clearly demonstrated that tau oligomers are not the direct therapeutic target of MB or LMTM.

In patients with AD, tau oligomers are detected at the early stages of pathogenic cascades^14–16^. Therefore, preventing disulfide tau oligomerization is an important therapeutic strategy to prevent neuronal loss and memory deficits in patients with AD. However, it has been difficult to establish a therapeutic strategy that prevents tau oligomerization since tau pathology is linked with a number of cellular processes that are closely linked to each other. For example, if a major tau kinase called GSK3β is inhibited, other tau kinases activate tau pathology^5,88^. Therefore, directly modifying tau would be a more effective strategy to prevent tau oligomerization without altering other cellular processes. Our results showed that levosimendan covalently binds to tau cysteine residues and inhibits tau oligomerization, preventing tau pathology. Moreover, levosimendan could disassemble tau-tau interactions regardless of its aggregation state. The binding mode between levosimendan and tau; how levosimendan dissociates disulfide-linked tau oligomers into monomers; and whether levosimendan can distinguish cytosolic tau from microtubule-bound tau should be clarified in future studies. Once levosimendan disassembles tau oligomers into monomers, monomeric tau is degraded by proteasome complexes, reducing the soluble tau burden^89^. Our results supported this, as the level of tau in levosimendan-treated tau-BiFC cells and primary neurons decreased, and the level of tau oligomers was reduced. Levosimendan displayed robust potency against tau oligomerization and rescued tauopathy-induced cognitive declines in the Tau^P301L^-BiFC mouse model. Although careful validation is needed, our data present the potential of levosimendan as a disease-modifying drug for AD.

## Supplementary information

Supplementary information
