# Supplementary material for: Levosimendan inhibits disulfide tau oligomerization and ameliorates tau pathology in TauP301L-BiFC mice
Source: Exp Mol Med. 2023 Mar 13;55(3):612–27. doi: 10.1038/s12276-023-00959-5 (PMC10073126; doi:10.1038/s12276-023-00959-5)
Supplement: Supplementary file 1 — Supplementary information [file 12276_2023_959_MOESM1_ESM.pdf]

Supplementary Information

Levosimendan inhibits disulfide tau oligomerization and ameliorates tau pathology in Tau^{P301L}-BiFC mice

Supplementary Fig. 1. Dose-dependent inhibition of tau-BiFC response activated by FK

Supplementary Fig. 2. Dose-dependent inhibition of tau-BiFC response activated by tauK18^{P301L}

Supplementary Fig. 3. Levosimendan inhibits tau oligomerization in tau-BiFC while MB and LMTM increased tau oligomerization.

Supplementary Fig. 4. Disulfide-linked tau oligomers in the human brain of AD and age-matched control (Non-AD)

Supplementary Table 1. Information on brain tissue samples from normal subjects and AD patinets

Supplementary Fig. 5. Effects of MB, LMTM and Levosimendan on tau kinases

Supplementary Fig. 6. Anti-tau aggregation effect of levosimendan treated at various time point after tau aggregation

Supplementary Fig. 7. Reaction of levosimendan with *N*-acetyl-L-cysteine methyl ester monitored by ^{1}H NMR

Supplementary Fig. 8. Disulfide-linked tau oligomers in wild type and Tau^{P301L} transgenic mice

Supplementary Data file 1. Characterization of ^{14}C-Levosimendan

Supplementary Data file 2. ESI-MS analysis of tau peptides

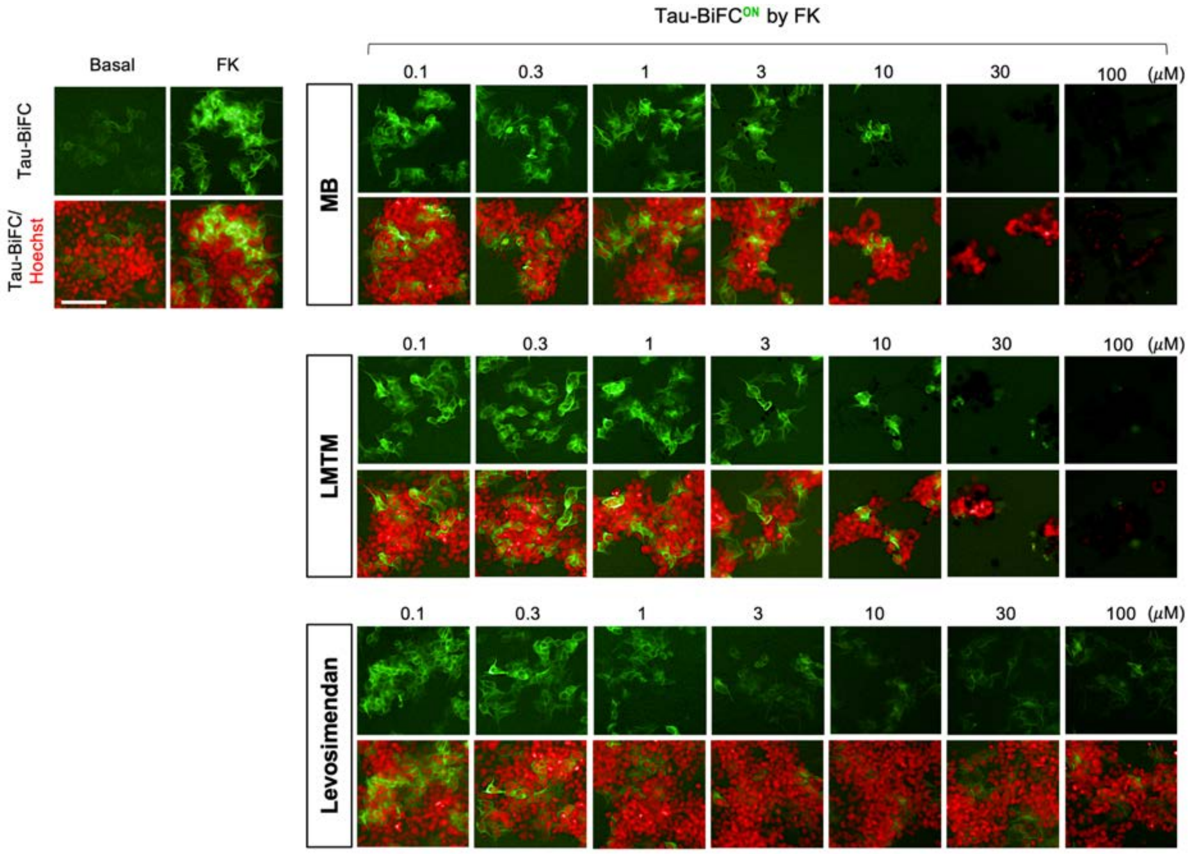

Supplementary Fig. 1 Dose-dependent inhibition of tau-BiFC response activated by FK. Tau-BiFC cells were incubated with MB, LMTM or levosimendan at various concentrations (0.1, 0.3, 1, 3, 10, 30, 100 μM) upon the activation with forskolin (30 μM) for 46 hrs. Nuclei were counterstained with Hoechst (red). Scale bar, 100 μm.

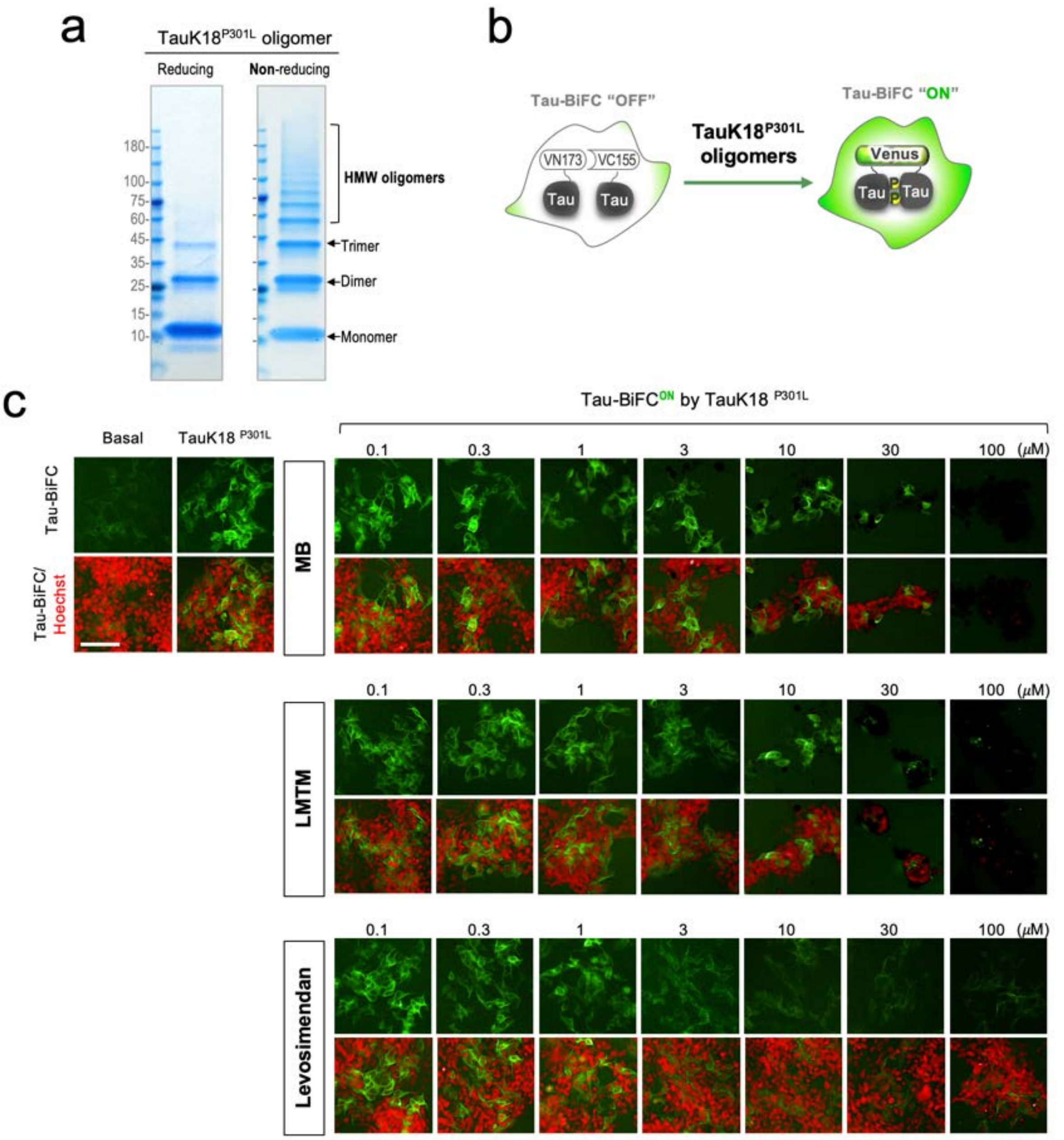

Supplementary Fig. 2 Dose-dependent inhibition of tau-BiFC response activated by tauK18^{P301L}.

(a) Reducing and non-reducing SDS-PAGE analysis of the tauK18^{P301L} oligomers. TauK18^{P301L} protein dissolved in PBS was separated on an SDS-PAGE gel (4-20%) under reducing or non-reducing condition. Tau oligomers on SDS-PAGE gels were visualized with Coomassie blue stain. Black arrow indicates tau monomer (14 kDa), dimer (28 kDa), and trimer (42 kDa). Bands above 45 kDa indicate high-molecular weight (HMW) tau oligomers. (b) Schematic diagram of the treatment of tauK18^{P301L} oligomers to tau-BiFC cells. (c) Tau-BiFC cells were incubated with MB, LMTM or levosimendan at various concentrations (0.1, 0.3, 1, 3, 10, 30, 100 μM) upon the activation with tauK18^{P301L} (5 μg/mL) for 46 hrs. Nuclei were counterstained with Hoechst (red). Scale bar, 100 μm.

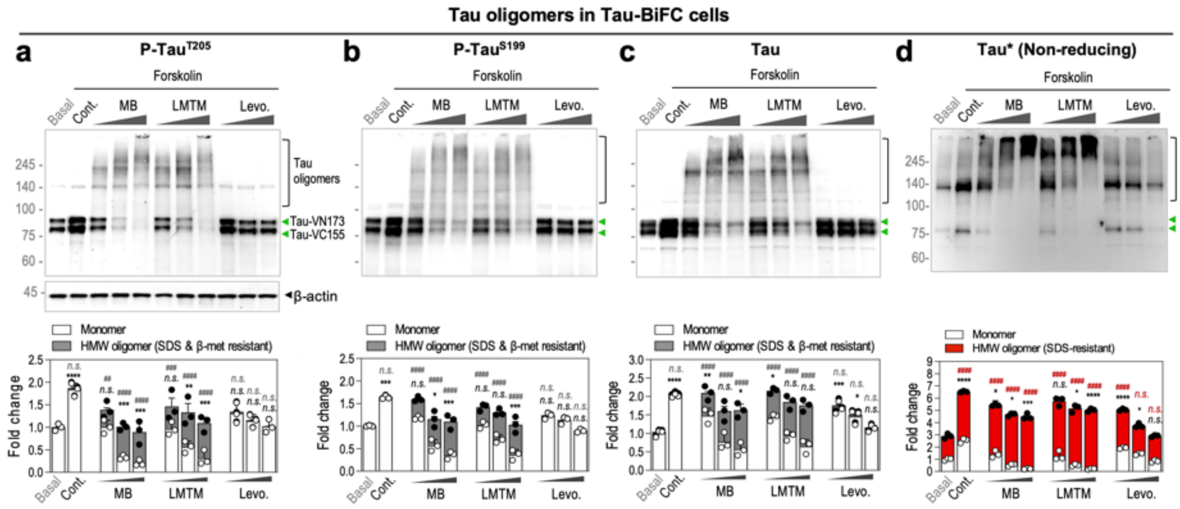

Supplementary Fig. 3 Levosimendan inhibits tau oligomerization in tau-BiFC while MB and LMTM increased tau oligomerization.

(a-c) Immunoblot analysis of total and phospho-tau. For the immunoblot analysis, tau-BiFC cells were treated with MB, LMTM (0.5, 1.5, 5 µM), or levosimendan (5, 15, 45 µM) in the presence of forskolin. Green arrows indicate monomer bands of hTau-VN173 and hTau-VC155. Bands above 100 kDa indicates tau oligomers. Relative amounts of monomeric tau (white bars) and oligomeric tau (grey bars) were quantified by Image J. β-actin is used as loading control. All data were normalized to β-actin. **(d)** Immunoblot analysis of total tau on non-reducing condition. Relative amounts of monomeric tau (white bars) and oligomeric tau (red bars) were quantified by Image J. **(a-d)** Data represent the mean ± S.D. of three independent experiments. To indicate significance of the results, Two-way ANOVA with Dunnett's multiple-comparisons test was performed; $^{*}p < 0.05$, $^{**}p < 0.01$, $^{***}p < 0.001$, $^{****}p < 0.0001$, compared with the level of monomer in basal. $^{\#}p < 0.05$, $^{\#\#}p < 0.01$, $^{\#\#\#}p < 0.001$, $^{\#\#\#\#}p < 0.0001$, compared with the level of oligomers in basal. *n.s.*, non-significant.

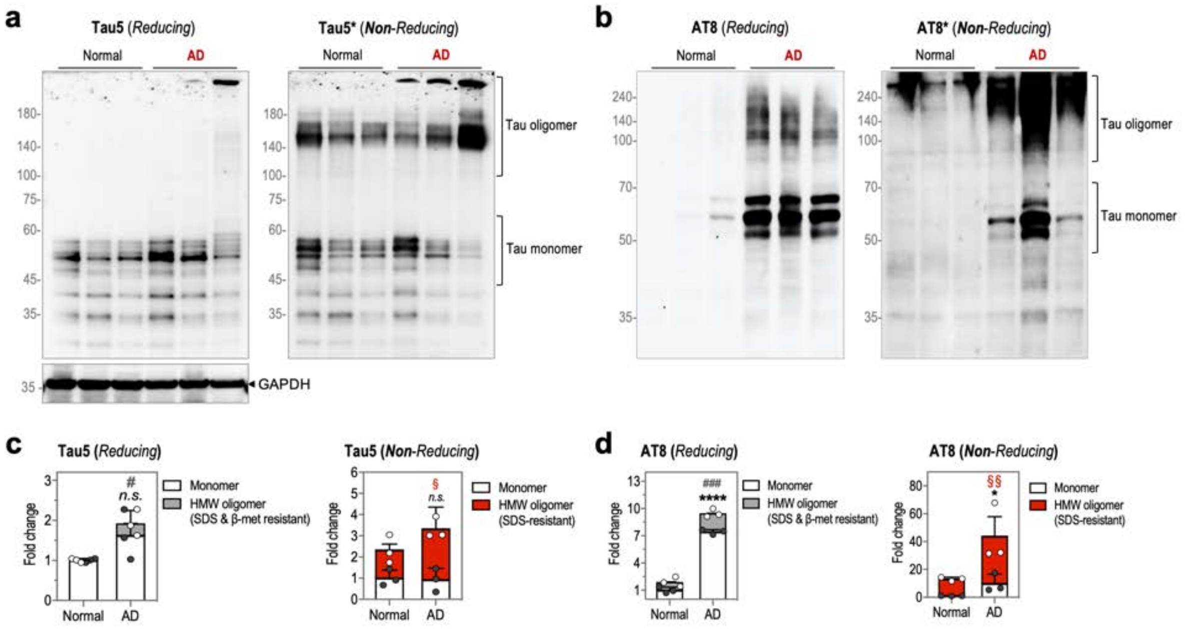

Supplementary Fig. 4 Disulfide-linked tau oligomers in the human brain of AD and age-matched control (Non-AD) (**a, b**) Total tau and phospho-tau immunoblot analysis of AD and Non-AD (normal) patients brain lysates. The brain lysates of AD and normal were separated under reducing and non-reducing conditions. GAPDH is used as loading control. All data were normalized to GAPDH. (**c, d**) Quantification of total tau and AT8-positive tau proteins. Relative amounts of tau monomers (white bars) and tau oligomers (red and gray bars) were quantified by Image J. A two-tailed *t*-test was performed; $^{****}p < 0.0001$ compared with tau monomer of normal group; $^{\S}p < 0.05$, $^{\S\S}p < 0.01$, $^{\#}p < 0.05$, $^{\#\#\#}p < 0.001$ compared with HMW oligomer of normal group.

Supplementary Table 1. Information on brain tissue samples from normal subjects and AD patients.

Number	Case	Age	Sex	Braak stage	PMI (Post-mortem interval)
1	Normal	87	F	I	4 h
2	Normal	86	M	II	3 h
3	Normal	78	F	I	6 h
4	AD	88	M	VI	7 h
5	AD	82	M	V	0.5 h
6	AD	79	F	VI	4 h

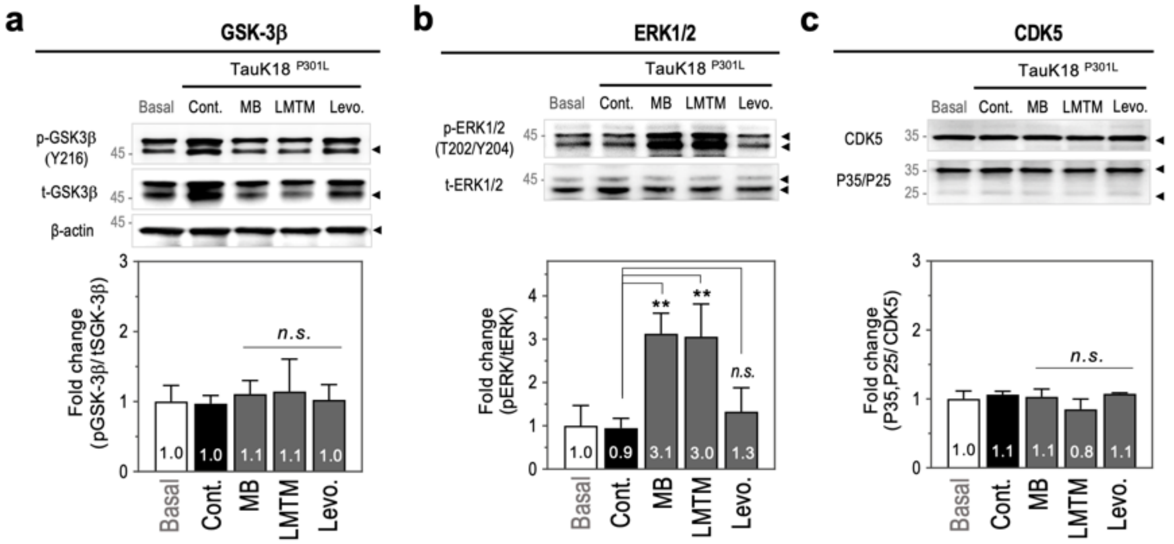

Supplementary Fig. 5 Effects of MB, LMTM and Levosimendan on tau kinases.

(a-c) Immunoblot analysis of tau kinases with anti-GSK-3β, anti-ERK1/2, anti-P35/P25, and anti-CDK5 antibodies. For the immunoblot analysis, primary neurons were treated with MB, LMTM (0.5 µM), or levosimendan (10 µM) in the presence of tauK18^{P301L}. β-actin is used as loading control. Relative amounts were quantified by Image J. All data were normalized to β-actin. One-way ANOVA with Dunnett's multiple-comparisons test was performed; $*p < 0.05$, $**p < 0.01$, $***p < 0.001$, compared with the level of control. *n.s.*, non-significant.

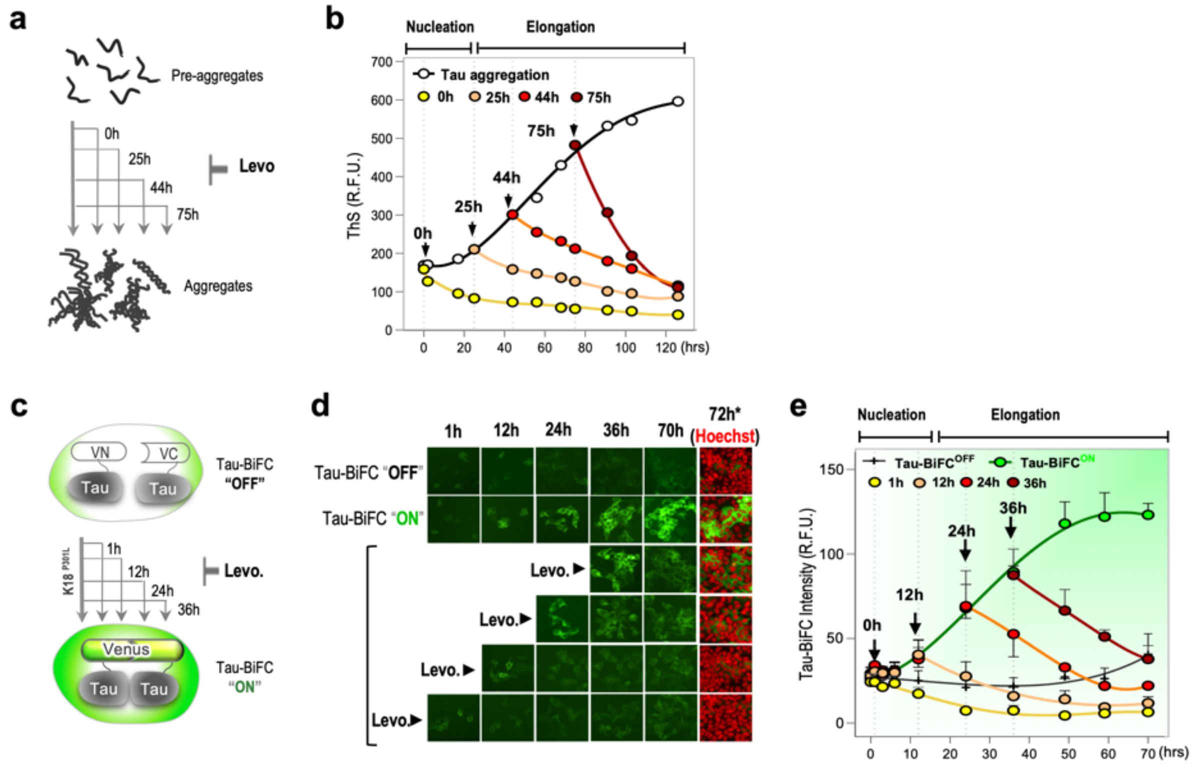

Supplementary Fig. 6 Anti-tau aggregation effect of levosimendan treated at various time points after tau aggregation.

(**a**) Schematic diagram of the treatment of levosimendan at diverse time points of tau aggregation *in vitro*. Levosimendan (10 µM) was treated to an aliquot of the aggregation mixture at 0, 25, 44, and 75hr after heparin treatment. The level of tau aggregation was determined by ThS. (**b**) ThS-response curves indicating anti-tau aggregation effect of levosimendan *in vitro*. (**c**) Schematic diagram of the treatment of levosimendan at various time points of tau-BiFC aggregation. Levosimendan was treated to tau-BiFC cells at 1, 12, 24, and 36 hrs after the treatment of tauK18^{P301L}. (**d**) Tau-BiFC fluorescence images were acquired at various time points and the fluorescence intensities were quantified. After 72hrs, nuclei were counterstained with Hoechst (red). Scale bar, 100 µm. (**e**) Tau-BiFC responses indicating the inhibitory effect of levosimendan on cellular tau aggregation. Data represent the mean ± S.D. of four repeated measurements.

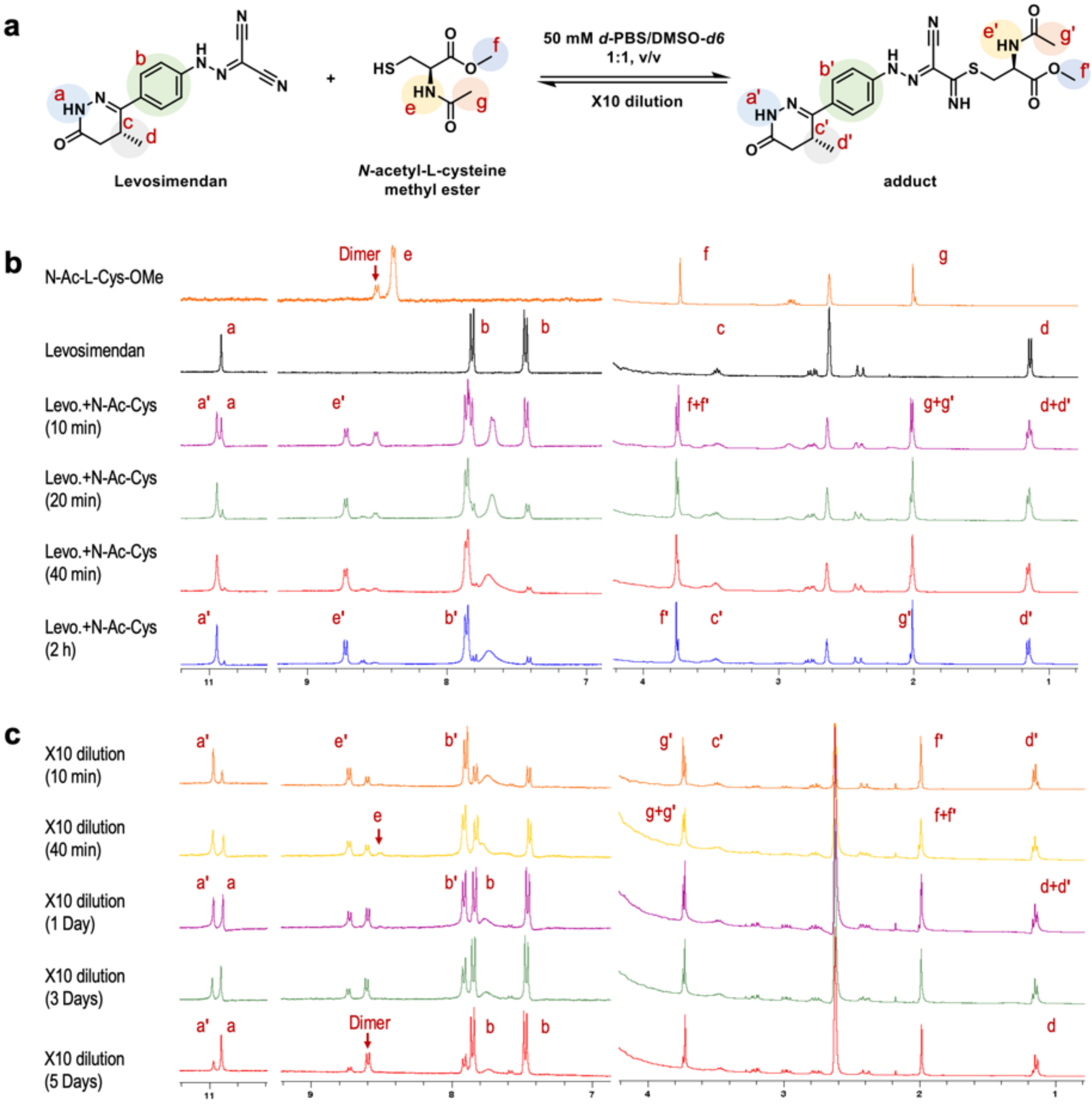

Supplementary Fig. 7 Reaction of levosimendan with *N*-acetyl-L-cysteine methyl ester monitored by ^{1}H NMR. (**a**) Proposed reaction scheme between levosimendan and *N*-acetyl-L-cysteine methyl ester. (**b**) Time-dependent display of ^{1}H NMR spectra of the reaction of levosimendan (180 μM, 1 equivalent) with *N*-acetyl-L-cysteine methyl ester (210 μM, 1.2 equivalent) in DMSO-d_6/d-PBS (1:1, v/v) at 25 °C. (**c**) Time-dependent display of ^{1}H NMR spectra of the reverse reaction upon ten times dilution of the reaction mixture with DMSO-d_6/d-PBS (1:1, v/v) at 25 °C. The ^{1}H NMR spectra of levosimendan and *N*-acetyl-L-cysteine methyl ester were taken in DMSO-d_6/D_2O (1:1, v/v) at 25 °C.

: Upon addition of *N*-acetyl-L-cysteine methyl ester to levosimendan, new proton peaks appeared indicating the generation of levosimendan-cysteine adduct. After 20 min, the ratio of levosimendan was reduced, presenting the decreasing proton peaks of 4,5-dihydropyridazin-3(2*H*)-one moiety H**a** (10.82 ppm), H**c** (~3.46 ppm), H**d** (~1.14 ppm), and benzene ring protons (H**b**, ~7.2~8.0 ppm) of levosimendan. At the 40 min time point, the adduct was steadily formed along with the production of a small amount of *N*-acetyl-L-cysteine methyl ester dimer (~8.6 ppm). After 2 hrs, the reaction was completed as the adduct was formed with corresponding

proton peaks H**a′** (10.94 ppm), (H**b′**, ~7.4~8.0 ppm), H**c′** (~3.46 ppm), H**d′** (~1.15 ppm) of levosimendan moiety and proton peaks of cysteine moiety including H**e′** (~8.7 ppm, amide NH), H**f′** (~3.7 ppm, methyl ester CH_3), and H**g′** (~2 ppm, acetyl CH_3). To evaluate the reversibility of the reaction, the reaction mixture was diluted ten times to promote *N*-acetyl-L-cysteine methyl ester dissociation (Fig S7c). Upon dilution, cysteine-dissociation was proceeded slowly and levosimendan was almost completely restored after 5 days, indicating reversible binding of levosimendan to the cysteine-thiol. While levosimendan was restored, *N*-acetyl-L-cysteine methyl ester dimer was generated as time passed.

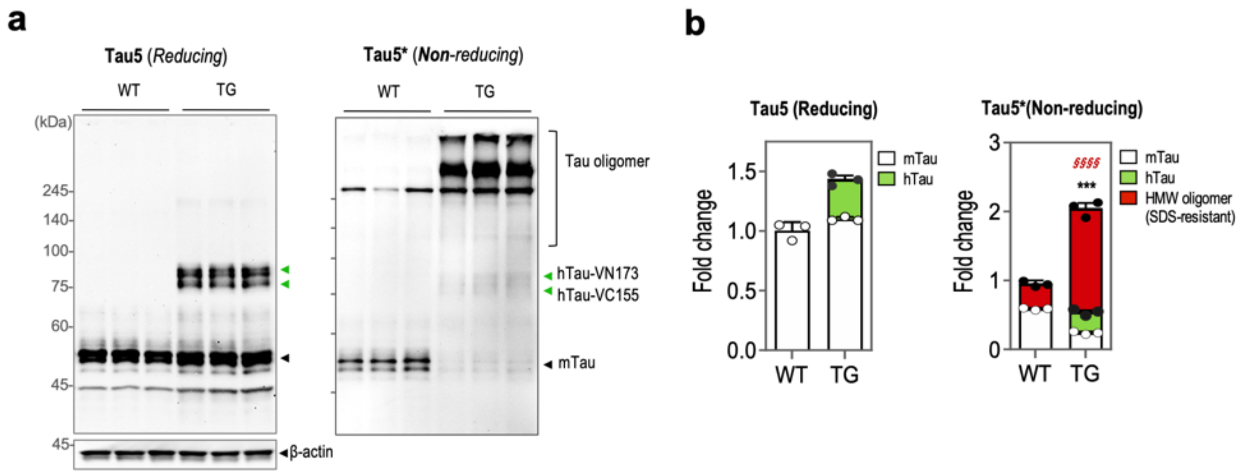

Supplementary Fig. 8 Disulfide-linked tau oligomers in wild type and Tau^{P301L} transgenic mice.

(a) Tau-immunoblot analysis of WT- and TG- brain lysates. RIPA-soluble fractions of wild type and Tau^{P301L}-BiFC mice (14-month-old) were separated under reducing and non-reducing conditions. Green arrows indicate hTau-VN173 and hTau-VC155. Black arrows indicate endogenous murine tau (mTau). β-actin is used as loading control. **(b)** Quantification of total tau protein (n=3 per group). Relative amounts of hTau (green bars), mTau (white bars) monomers, and oligomers (red bars) were quantified by Image J. All data were normalized to β-actin. A two-tailed *t*-test was performed; ***$p < 0.001$, compared with mTau monomer of wild type; §§§§$p < 0.0001$, compared with HMW oligomer of wild type mice.

Supplementary Data file 1. Characterization of ^{14}C-Levosimendan

a ^{1}H NMR spectrum of Levosimendan.

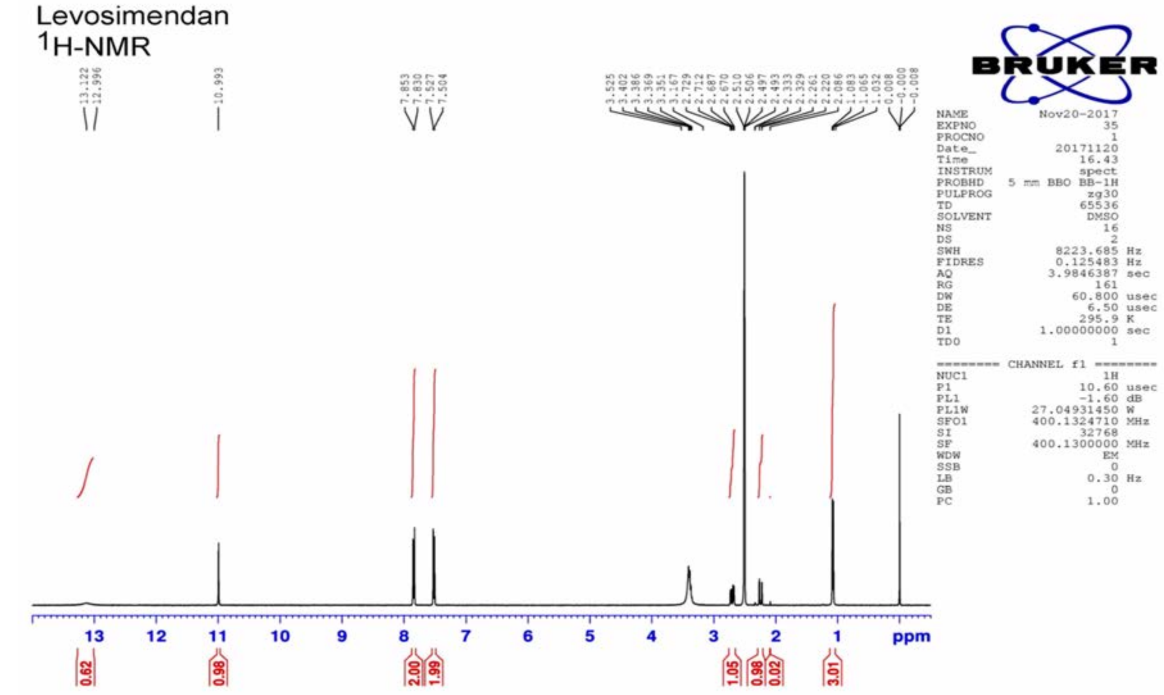

b ^{1}H NMR spectrum of ^{14}C-Levosimendan.

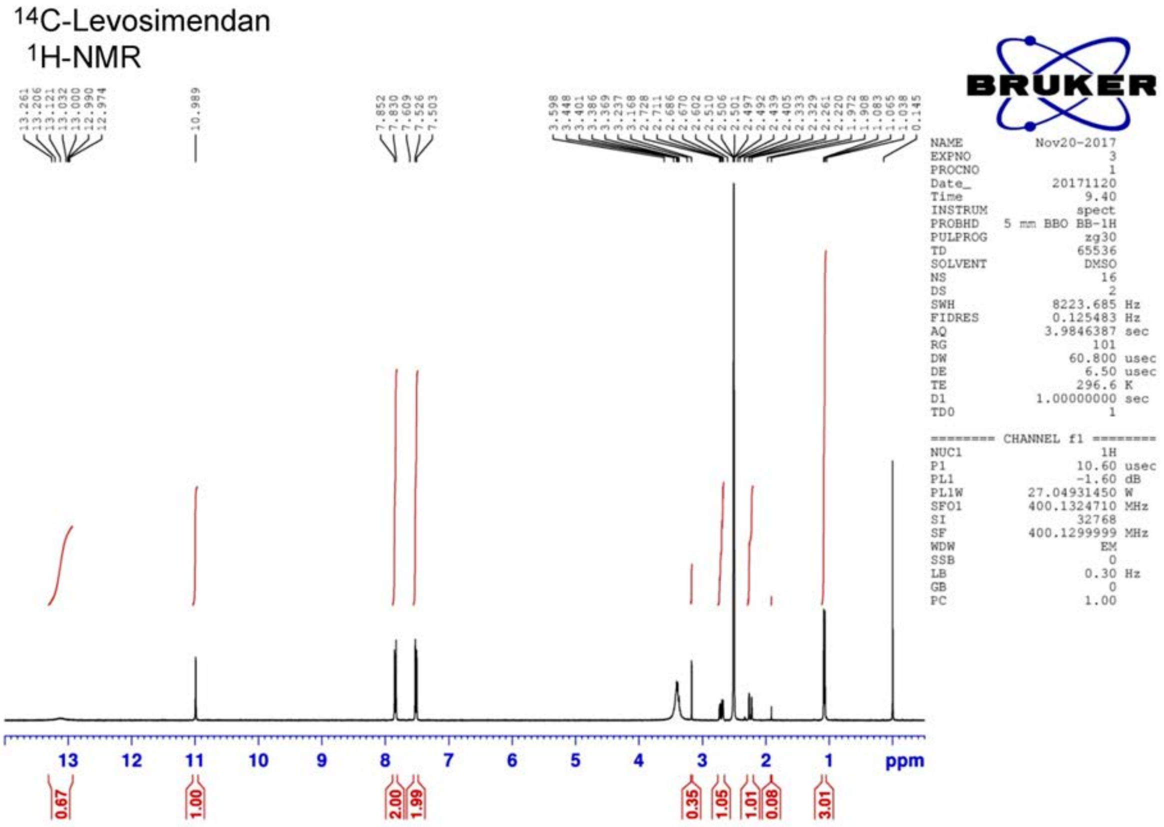

c LC/MS characterization of ^{14}C-Levosimendan

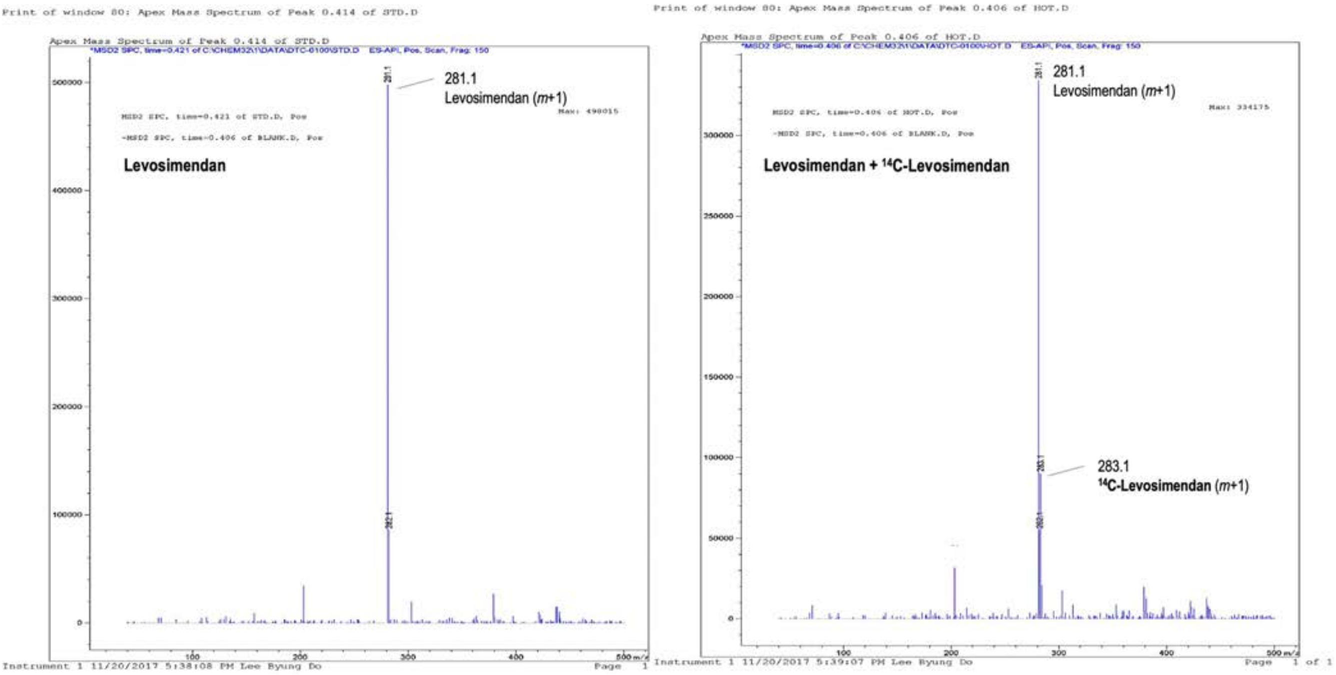

Supplementary Data file 2. ESI-MS analysis of tau peptides

a ESI-MS analysis of tau repeat 1 domain (Tau R1).

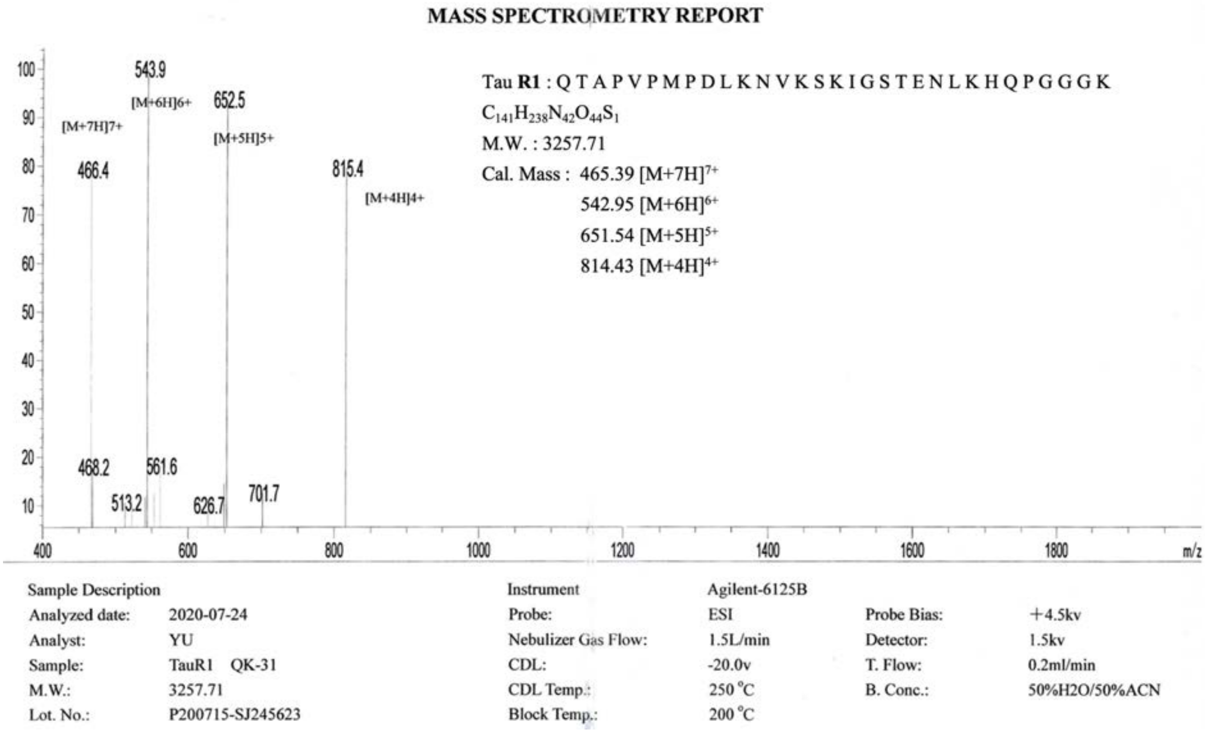

b ESI-MS analysis of tau repeat 2 domain (Tau R2).

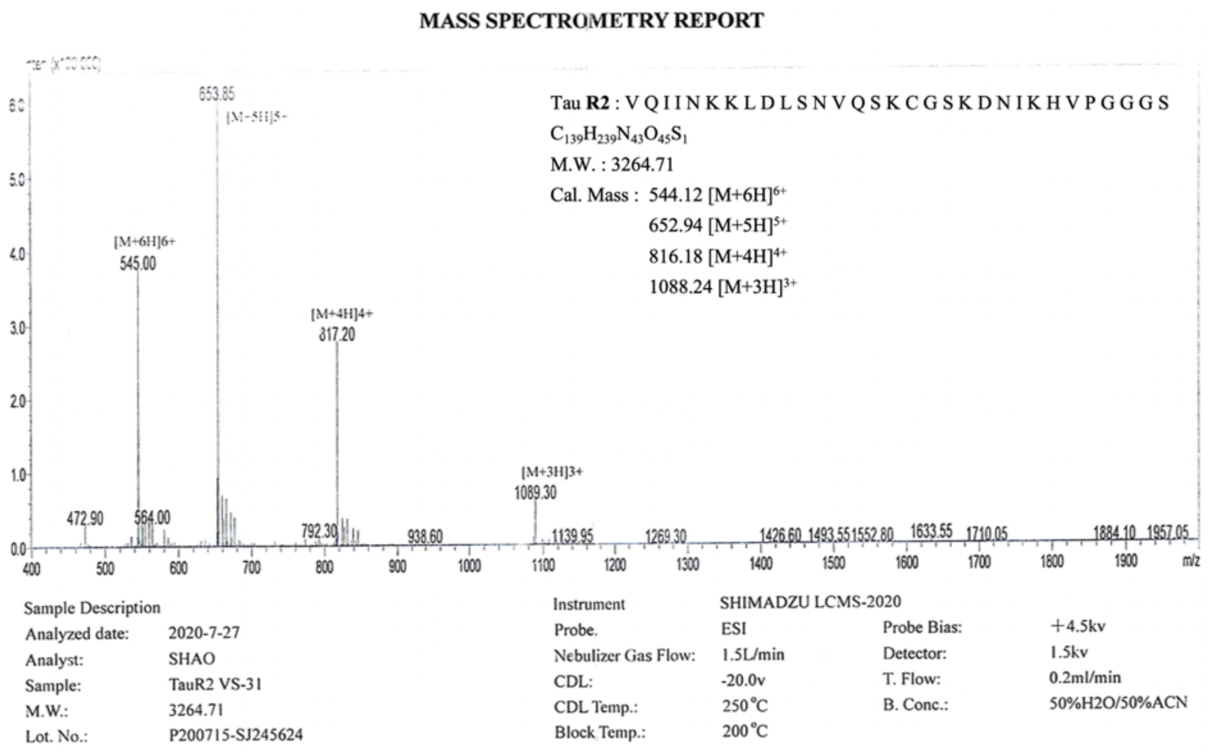

c ESI-MS analysis of tau repeat 3 domain (Tau R3).

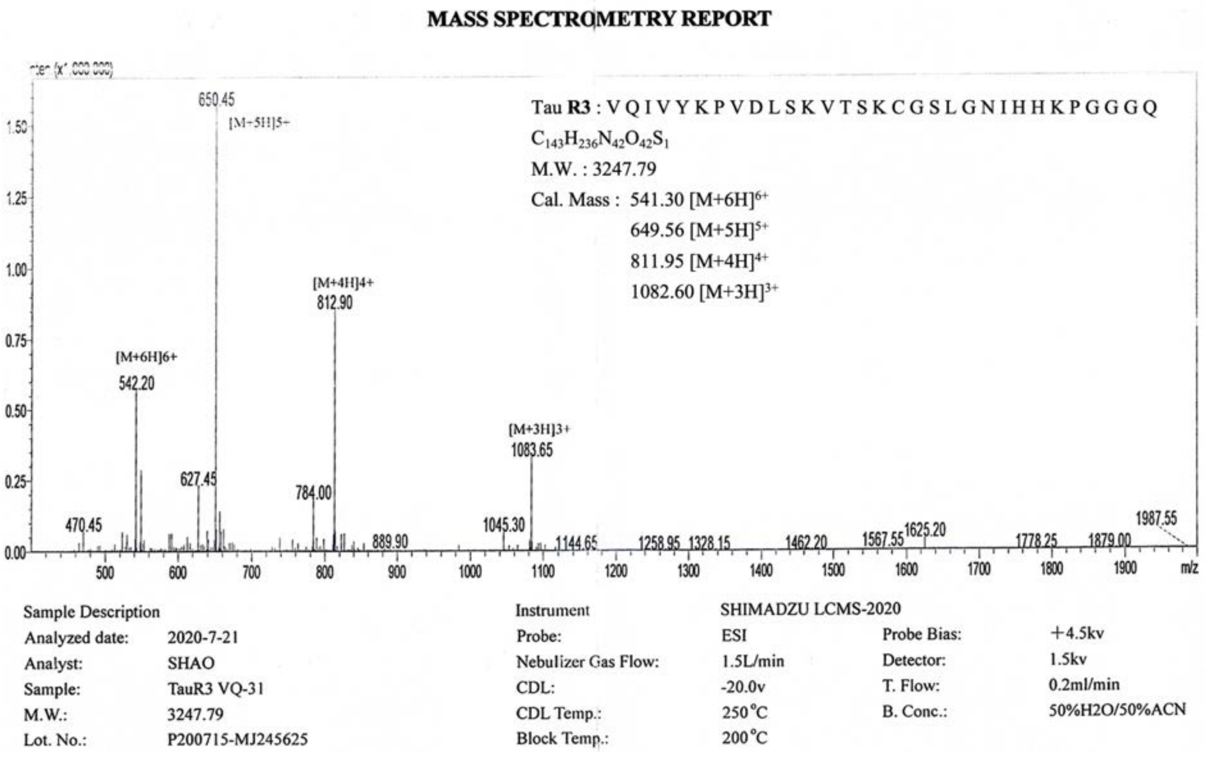

d ESI-MS analysis of tau repeat 4 domain (Tau R4).

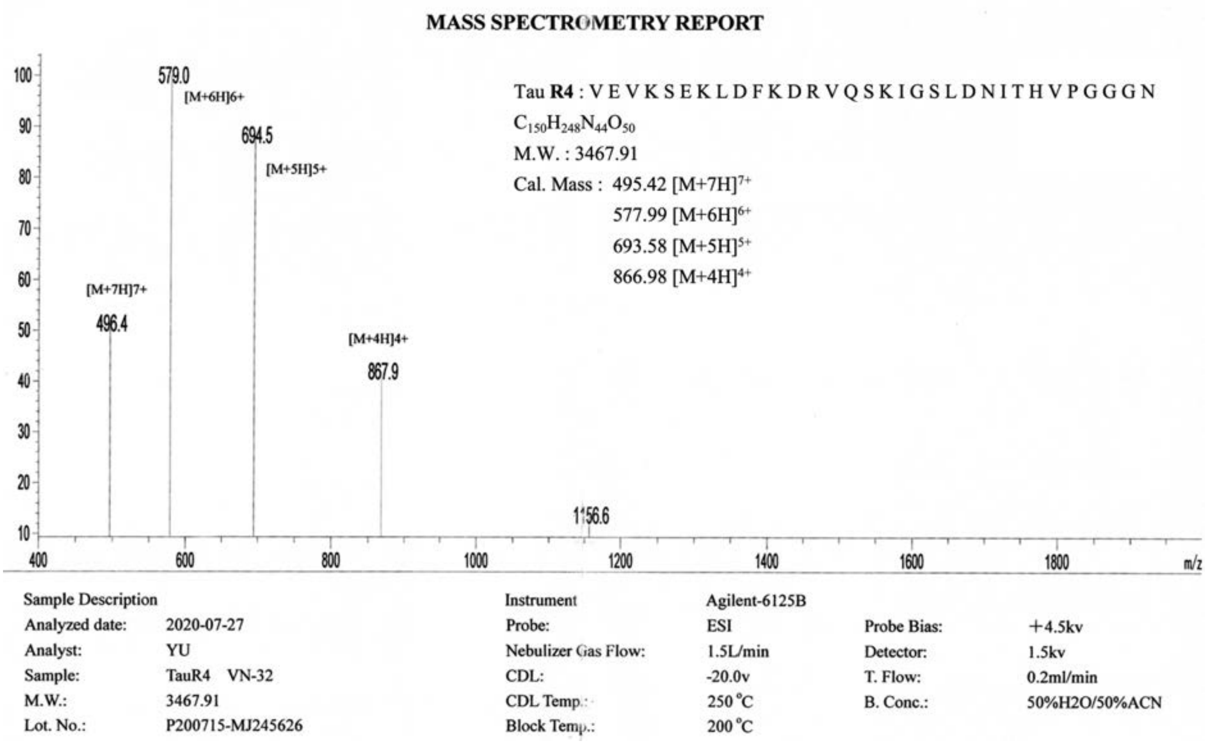

e ESI-MS analysis of tau repeat 2 domain including cysteine mutant (Tau R2C291S).

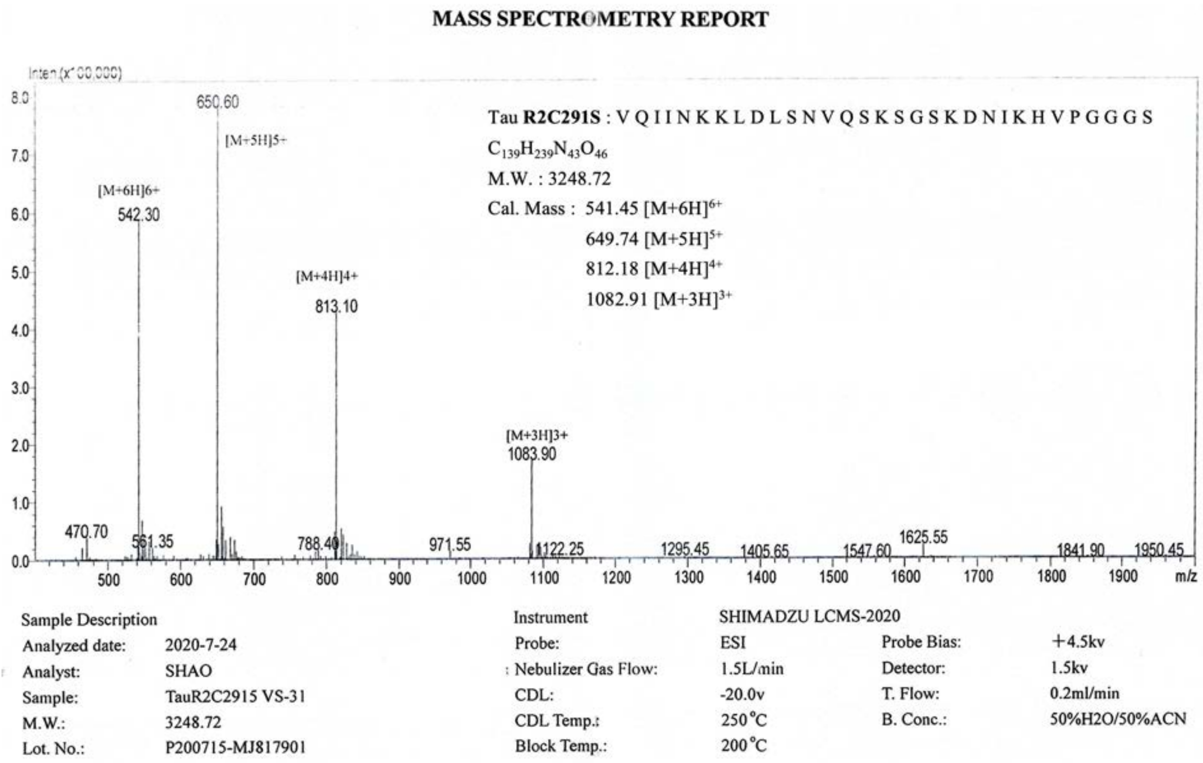

f ESI-MS analysis of tau repeat 3 domain including cysteine mutant (Tau R3C322S).

MASS SPECTROMETRY REPORT

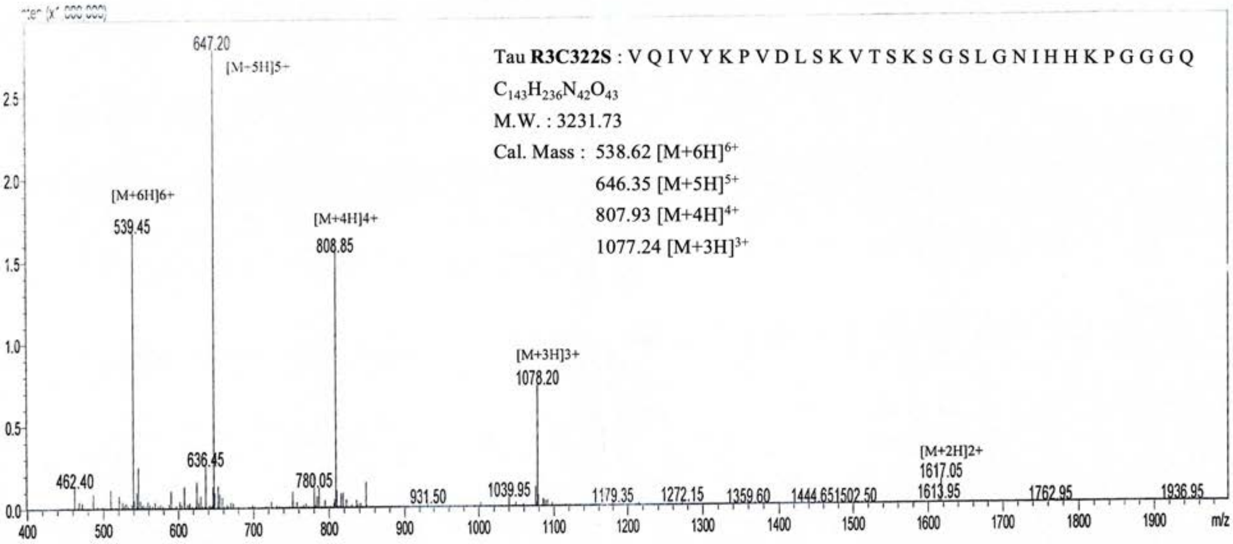

Sample Description		Instrument	SHIMADZU LCMS-2020		
Analyzed date:	2020-7-23	Probe:	ESI	Probe Bias:	+4.5kv
Analyst:	SHAO	Nebulizer Gas Flow:	1.5L/min	Detector:	1.5kv
Sample:	TauR3C322S VQ-31	CDL:	-20.0v	T. Flow:	0.2ml/min
M.W.:	3231.73	CDL Temp.:	250°C	B. Conc.:	50%H2O/50%ACN
Lot. No.:	P200715-MJ817902	Block Temp.:	200°C		
